# Supplementary material for: Maternal and food microbial sources shape the infant microbiome of a rural Ethiopian population
Source: Curr Biol. 2023 May 22;33(10):1939–1950.e4. doi: 10.1016/j.cub.2023.04.011 (PMC10234599; doi:10.1016/j.cub.2023.04.011)
Supplement: Document S1. Figures S1 and S2 [file mmc1.pdf]

Current Biology, Volume *33*

Supplemental Information

Maternal and food microbial sources

shape the infant microbiome

# of a rural Ethiopian population

Serena Manara, Marta Selma-Royo, Kun D. Huang, Francesco Asnicar, Federica Armanini, Aitor Blanco-Miguez, Fabio Cumbo, Davide Golzato, Paolo Manghi, Federica Pinto, Mireia Valles-Colomer, Loredana Amoroso, Maria Valeria Corrias, Mirco Ponzoni, Roberta Raffaetà, Raul Cabrera-Rubio, Mari Olcina, Edoardo Pasolli, Maria Carmen Collado, and Nicola Segata

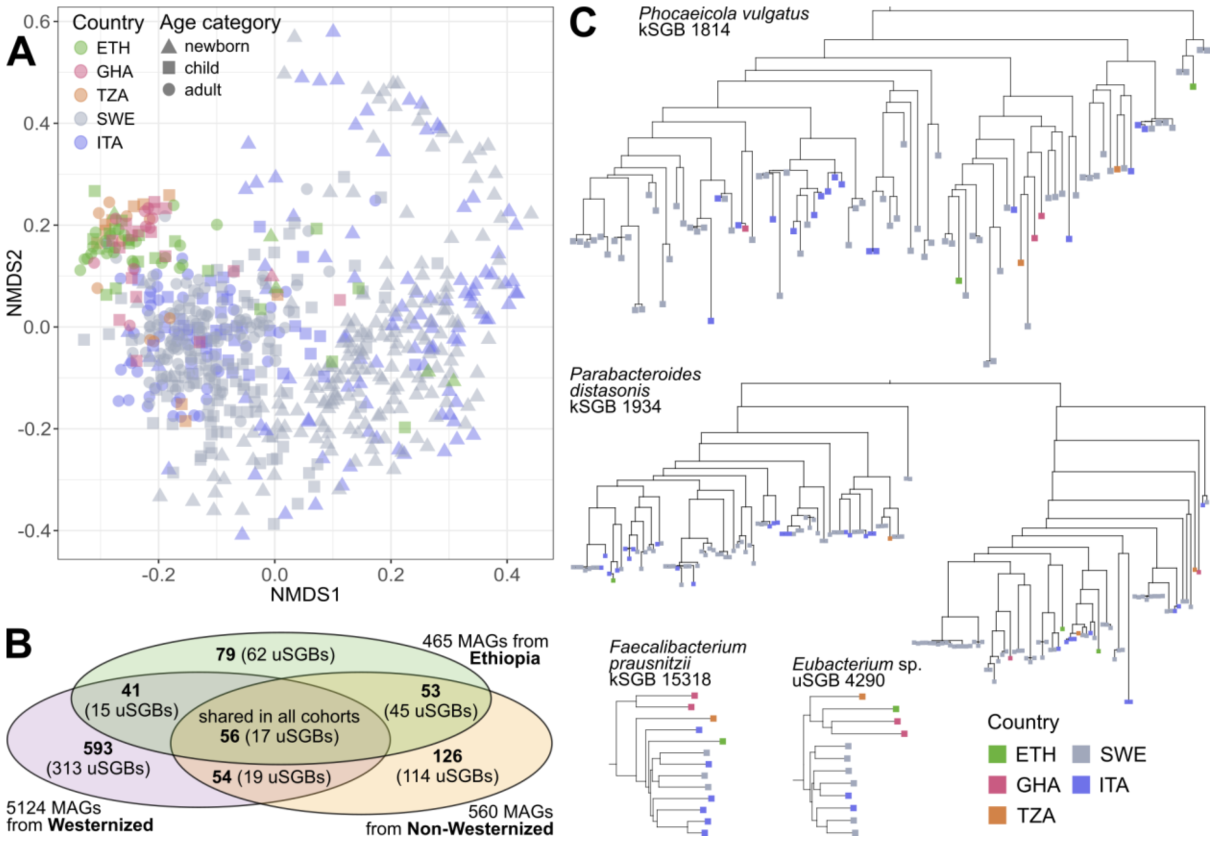

Figure S1. Ethiopian mother and infant microbiome composition differs from that of Italian and Swedish age-matched populations and is more similar to the one of Tanzanian and Ghanaian ones. Related to Figure 1. A) Multidimensional scaling of the Bray-Curtis distance on sample taxonomic composition shows a trend ranging from newborns (triangles, <1 year) to children (squares, >1 year) and adults (circles, >18 years). Samples with single species relative abundance > 90% have been removed. **B)** SGB sharing across different infant cohorts. Differently from what is reported in **Figure 1C**, here an SGB is considered shared between Ethiopia and non-Westernized (or Westernized) cohorts even if it is present only in one of the two countries part of that specific lifestyle category. Even in this case, Ethiopian infants share a larger proportion of SGBs and particularly uSGBs with infants from other Non-Westernized cohorts (26.7% of the total number of SGBs found in the Ethiopian and non-Westernized infants) and have an even more limited SGB overlap with Westernized infants microbiomes (11.1% of of the total number of SGBs found in the Ethiopian and Westernized infants). **C)** Phylogenetic trees of additional relevant species shared across Ethiopian, Westernized, and Non-Westernized infants, including two further SGBs of the same species reported in **Figure 1D**.

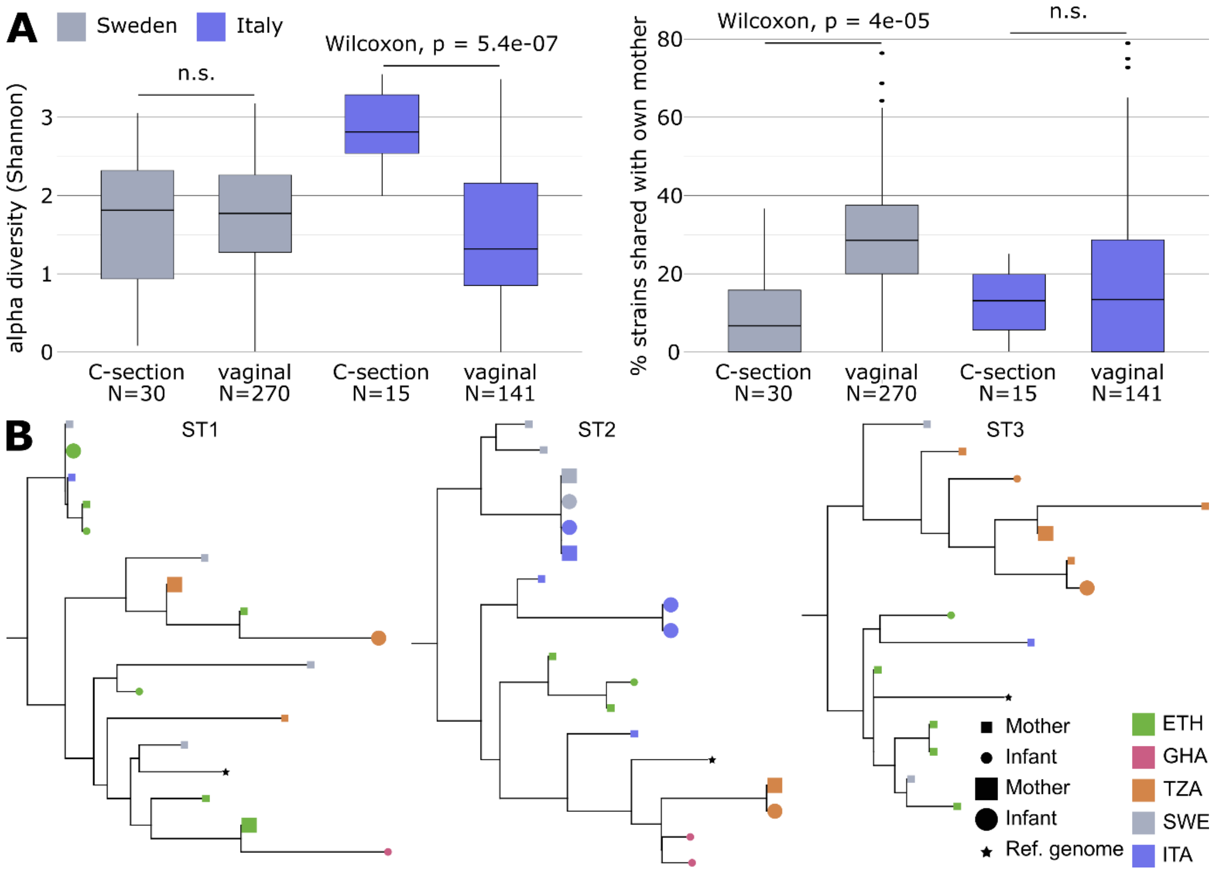

Figure S2. Potential modifiers of strain-sharing rate. Related to Figures 2 and 3. A) Differences in alpha diversity (Shannon) and strain sharing with own mother by mode of delivery for the two cohorts that include samples from C-section delivered infants (Sweden 30/300, Italy 15/156). **B)** Phylogenetic trees of *Blastocystis* sp. STs present also in Ethiopian samples (ST1, ST2, ST3). ST2 shows the highest degree of mother-infant sharing, with 4 out of 4 positive pairs showing the presence of the same strain. ST1 and ST3, instead, were not shared at the strain level between mother and infant.
