# Supplementary material for: Maternal and food microbial sources shape the infant microbiome of a rural Ethiopian population
Source: Curr Biol. 2023 May 22;33(10):1939–1950.e4. doi: 10.1016/j.cub.2023.04.011 (PMC10234599; doi:10.1016/j.cub.2023.04.011)
Supplement: Document S2. Article plus supplemental information [file mmc8.pdf]

Current Biology

Article

Maternal and food microbial sources shape the infant microbiome of a rural Ethiopian population

Highlights

- Microbiome sharing is reduced in Ethiopian and non-westernized mother-infant pairs
- Some uncharacterized species are present and shared only in the Ethiopian cohort
- Locally produced fermented food provides some of these unusual microbiome species
- The microbiome is impacted by lifestyle and also through mother-infant strain-sharing patterns

Authors

Serena Manara, Marta Selma-Royo, Kun D. Huang, ..., Edoardo Pasolli, Maria Carmen Collado, Nicola Segata

Correspondence

edoardo.pasolli@unina.it (E.P.), mcolam@iata.csic.es (M.C.C.), nicola.segata@unitn.it (N.S.)

In brief

Manara et al. investigate how lifestyle affects mother-infant microbiome sharing. Manara et al. show that Ethiopian and other non-westernized infants share a smaller microbiome fraction (mainly uncharacterized species) with their mothers and identify some unusual mother-infant shared species present in locally produced fermented food.

Manara et al., 2023, Current Biology *33*, 1939–1950
May 22, 2023 © 2023 The Authors. Published by Elsevier Inc.
https://doi.org/10.1016/j.cub.2023.04.011

Current Biology

CellPress
OPEN ACCESS

Article

Maternal and food microbial sources shape the infant microbiome of a rural Ethiopian population

Serena Manara,[1,8] Marta Selma-Royo,[1,2] Kun D. Huang,[1] Francesco Asnicar,[1] Federica Armanini,[1] Aitor Blanco-Miguez,[1] Fabio Cumbo,[1] Davide Golzato,[1] Paolo Manghi,[1] Federica Pinto,[1] Mireia Valles-Colomer,[1] Loredana Amoroso,[3] Maria Valeria Corrias,[4] Mirco Ponzoni,[4] Roberta Raffaetà,[5] Raul Cabrera-Rubio,[2] Mari Olcina,[6] Edoardo Pasolli,[7,9,*] Maria Carmen Collado,[2,10,*] and Nicola Segata[1,11,12,13,*]

[1]Department of Cellular Computational and Integrative Biology, Via Sommarive 9, Povo, Trento 38123, Italy
[2]Institute of Agrochemistry and Food Technology- National Research Council (IATA-CSIC), C/ Catedrático Agustín Escardino Benlloch, 7, 46980 Paterna, Valencia, Spain
[3]Oncology Unit, IRCCS Istituto Giannina Gaslini, Via Gerolamo Gaslini 5, 16147 Genoa, Italy
[4]Laboratory of Experimental Therapies in Oncology, IRCCS Istituto Giannina Gaslini, Via Gerolamo Gaslini 5, 16147 Genoa, Italy
[5]Ca' Foscari University Venice, Department of Philosophy and Cultural Heritage and NICHE, Malcanton Marcorà, Dorsoduro 3484/D, 30123 Venice, Italy
[6]Department of Preventive Medicine and Public Health, Faculty of Pharmacy, Universitat de València, Av. Vicent Andrés Estellés s/n, Burjassot, Valencia 46100, Spain
[7]Department of Agricultural Sciences, University of Naples Federico II, Via Università 100, 80055 Portici, Naples, Italy
[8]Twitter: @SerenaManara
[9]Twitter: @epasolli
[10]Twitter: @mcarmen_collado
[11]Twitter: @nsegata
[12]Twitter: @cibiocm
[13]Lead contact
*Correspondence: edoardo.pasolli@unina.it (E.P.), mcolam@iata.csic.es (M.C.C.), nicola.segata@unitn.it (N.S.)
https://doi.org/10.1016/j.cub.2023.04.011

SUMMARY

The human microbiome seeding starts at birth, when pioneer microbes are acquired mainly from the mother. Mode of delivery, antibiotic prophylaxis, and feeding method have been studied as modulators of mother-to-infant microbiome transmission, but other key influencing factors like modern westernized lifestyles with high hygienization, high-calorie diets, and urban settings, compared with non-westernized lifestyles have not been investigated yet. In this study, we explored the mother-infant sharing of characterized and uncharacterized microbiome members via strain-resolved metagenomics in a cohort of Ethiopian mothers and infants, and we compared them with four other cohorts with different lifestyles. The westernized and non-westernized newborns' microbiomes composition overlapped during the first months of life more than later in life, likely reflecting similar initial breast-milk-based diets. Ethiopian and other non-westernized infants shared a smaller fraction of the microbiome with their mothers than did most westernized populations, despite showing a higher microbiome diversity, and uncharacterized species represented a substantial fraction of those shared in the Ethiopian cohort. Moreover, we identified uncharacterized species belonging to the *Selenomonadaceae* and *Prevotellaceae* families specifically present and shared only in the Ethiopian cohort, and we showed that a locally produced fermented food, injera, can contribute to the higher diversity observed in the Ethiopian infants' gut with bacteria that are not part of the human microbiome but are acquired through fermented food consumption. Taken together, these findings highlight the fact that lifestyle can impact the gut microbiome composition not only through differences in diet, drug consumption, and environmental factors but also through its effect on mother-infant strain-sharing patterns.

INTRODUCTION

The gut microbiome is an integral part of human health that starts to be acquired and shaped at birth. Substantial seeding of the newborn microbiome from the maternal microbiome occurs already at birth, with a strong influence of mode of delivery[1–5] and intrapartum or postpartum antibiotic prophylaxis,[6,7] and with potential long-term effects.[8,9] Breast- or formula-feeding is another factor that showed an effect in shaping the infant microbiome in the first months of life,[10–13] with weaning representing a turning point in gut microbiome maturation.[10,12,14,15] The initial colonization of the gut microbiome by microbes transmitted

© 2023 The Authors. Published by Elsevier Inc.
This is an open access article under the CC BY license (http://creativecommons.org/licenses/by/4.0/).

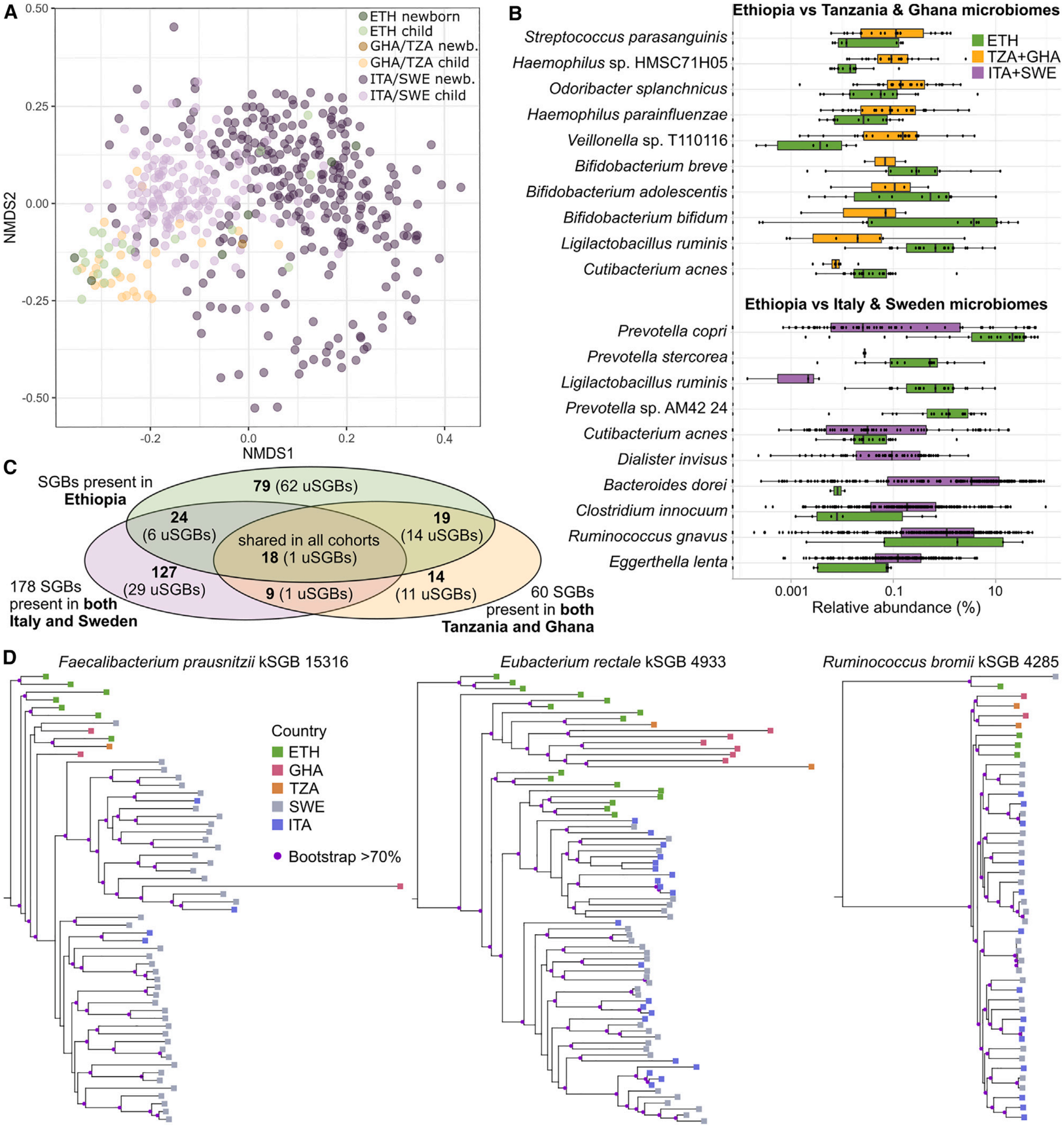

Figure 1. Ethiopian mother and infant microbiome composition differs from that of age-matched Italian and Swedish subjects but resembles that of the two Tanzanian and Ghanaian populations

(A) Non-metric multidimensional scaling of the Bray-Curtis distance on sample taxonomic composition shows a trend ranging from newborns (<1 year of age, darker colors) to children (≥1 year of age, lighter colors). Ethiopian samples (green shades) tend to cluster together and with samples from the Tanzanian and Ghanaian communities (yellow shades), with the exception of some infant and child samples that cluster with same-age samples of the Italian and Swedish cohorts (violet shades). NMDS presenting the different cohorts, including adult samples, is available in Figure S1A.

(B) Species most associated with Ethiopian, westernized (here, Italian and Swedish), and non-westernized (here, Tanzanian and Ghanaian) infants aged 0–12 years. Because of the different age distribution between non-westernized and westernized infants, we randomly picked westernized infants aged 0–6 months, 6–12 months, and 1–12 years to get a similar age distribution with respect to Ethiopian ones before performing a Wilcoxon test. The test was repeated 10 times with 10 different random pickings, with each test comparing the microbiome composition of 127 westernized and 20 Ethiopian infants (0–6 months: 55 West. and 12 Eth.; 6–12 months: 27 West. and 6 Eth.; 12–36 months: 9 West. and 2 Eth.). Non-westernized cohorts already had an age distribution similar to the Ethiopian one, so we performed a simple Wilcoxon test (28 non-West. and 26 Eth.). Reported here are the 20 species that showed the strongest association with Ethiopian,

(legend continued on next page)

from the mother at delivery and during the first months of life has been identified as a key factor in microbiome development, and different species have been identified as commonly shared between mother and child and retained over time.[10,15–17]

Several factors shape the microbiome during the whole life, including diet,[18–20] antibiotic use,[6,21] and other lifestyle aspects.[22,23] These factors have been investigated together in multiple studies that contrasted the human microbiome in populations living in highly industrialized countries, with limited contact with wildlife, high-calorie diets, higher exposure to xenobiotics, highly processed foods, antibiotics, and antimicrobials (westernized communities) against that of populations living in more rural areas with larger exposure to wildlife or domesticated animals, local food production and consumption, and limited access to pharmaceuticals (non-westernized communities).[22–25] However, no studies so far focused on how mother-to-infant transmission of the microbiome is impacted by the westernization process, which includes particularly controlled infant delivery settings and care. It is thus currently unknown how maternal and environmental seeding varies in populations with radically different lifestyles, and insights into this phenomenon could explain the different microbiome composition in westernized versus non-westernized populations that was extensively described for adult individuals. Expanding mother-to-infant microbiome transmission analysis with metagenomes from underrepresented community lifestyles would help characterizing the universal dynamics of microbiome structures and development.

In this study, we aimed to survey the potentially different dynamics of mother-infant strain sharing in westernized and non-westernized communities and to highlight the difference in regard to which species are shared. To this end, we assessed gut microbiome members strain sharing between mothers and their infants in an Ethiopian cohort[26] and compared the results with two westernized and two non-westernized age-matched cohorts[10,15,16,26] that we expanded with 70 newly sequenced stool metagenomes from healthy infants and their mothers living in northern Italy. Additional metagenomic sequencing of locally produced fermented foods (Ethiopian injera—fermented teff flour) were tested for their potential source of microbiome diversity for the Ethiopian mothers and infants enrolled in this study.

RESULTS

To investigate the mother-infant microbiome sharing patterns in rural non-westernized populations and to test the hypothesis that such patterns may differ from those in westernized populations, we analyzed here a metagenomic cohort of 25 mother-infant pairs from two villages in Ethiopia (Igu-kura and Gimbichu). For all the enrolled individuals, we performed metagenomic sequencing of the stool microbiome and characterized mother-infant sharing events via newly improved strain-resolved mapping-based metagenomic tools[27,28] as well as via metagenomic assembly. The same computational profiling was applied on a total of 580 samples from existing mother and infants cohorts spanning geographical location and distinct lifestyles[10,15,16,26] and further expanded with 70 newly sequenced stool samples obtained from age-matched healthy mother-infant pairs living in urban areas in northern Italy. We moreover collected and sequenced two samples of a typical fermented Ethiopian food (sourdough made with teff flour) that is part of the traditional postpartum diet and is used during weaning in the investigated cohort and could therefore be a potential source of mother and infant microbiome members. In total, we analyzed 702 metagenomic samples, including stool samples collected from newborns (here defined as infants <1 year of age), children (1–12 years old), and their mothers. In the present study, we use the term "infant" to refer to newborns and children as a single group.

The composition of the gut microbiome of Ethiopian children is distinct from that of westernized populations

Metagenomic sequencing and quantitative taxonomic profiling using MetaPhlAn 3 (see STAR Methods) showed that the microbiome composition of Ethiopian newborns highly overlaps with the one of other same-age individuals living in rural non-westernized communities from previous studies from Ghana and Tanzania[26] (PERMANOVA [permutational multivariate analysis of variance] p value newborns = 0.85), while only partially resembling the one of European newborns and children from Sweden[10] and Italy[15,16] (PERMANOVA p value = 0.003; Figure 1A). Importantly, the difference between Ethiopian and European infants increases with the age of the individuals, with newborns showing substantially less divergence than children and adults (Figure 1A), suggesting an effect of the milk-based diet that is common to all cohorts at a very young age. Accordingly, this difference in microbiome composition between Ethiopian and westernized populations increases in children probably as an effect of the difference in diet during and after weaning across lifestyles, and it becomes even clearer in adulthood, when all samples from non-westernized populations cluster tightly together and are well separated from those from westernized populations (PERMANOVA p value = 0.001, Figure S1A).

Several microbial species were specifically associated with the infants of the Ethiopian cohort when compared with those in westernized populations (Figure 1B). In particular, *Dialister*

westernized, or non-westernized infants and that had >0.05% average relative abundance and >20% prevalence in at least one of the two compared categories. For a full list, see Data S1. The box plots show the first and third quartiles (boxes) and the median (middle line); the whiskers extend up to 1.5× the IQR.

(C) Ethiopian infants share more species-level genome bins (SGBs, spanning < 5% genetic diversity, see STAR Methods) and particularly more uSGBs (SGBs assigned to uncharacterized species) with infants from Tanzania and Ghana (22.7% of SGBs found in Ethiopian and in both non-westernized infants' cohorts) and have only limited SGB overlap with Italian and Swedish infants' microbiomes (15.2% of SGBs found in Ethiopian and in both westernized infants' cohorts). Of these overlapping SGBs, only 18 are found at least once in infants from all countries, with only 1 uSGB shared across all cohorts (*Eubacterium* SGB4290; more details in Data S2C).

(D) Phylogenetic trees based on SGB-specific core genes (see STAR Methods) of the most relevant species shared across all infant cohorts included in this study (≥2 positive infants per cohort, SGBs taxonomically assigned to the same species are summed. Subtrees with bootstrap > 70% are identified with purple circles. The term kSGB indicates an SGB assigned to species for which reference genomes are available; uSGB indicates an SGB assigned to a species lacking reference genomes). Additional phylogenetic trees of interesting species are reported in Figure S1C.

See also Tables S1 and S4.

invisus, *Bacteroides dorei*, *Clostridium innocuum*, *Ruminococcus gnavus*, and *Eggerthella lenta* were associated with European samples (Wilcoxon rank-sum test with FDR-corrected p values < 0.05), whereas *Prevotella copri*, *Prevotella stercorea*, *Prevotella* sp. AM42 24, and *Ligilactobacillus ruminis* (previously *Lactobacillus ruminis*) were those most associated with Ethiopian microbiomes (Figure 1B; Data S1A). *L. ruminis* was associated with Ethiopian infant microbiomes even when compared with the other available non-westernized cohorts. The comparison between Ethiopian and the other two non-westernized populations highlighted three *Bifidobacterium* species, namely *B. breve*, *B. adolescentis*, and *B. bifidum*, that were more associated with Ethiopian infant microbiomes (Figure 1B; Data S1B). On the other hand, Ghanaian and Tanzanian infant microbiomes were more associated than Ethiopian ones with *Streptococcus parasanguinis*, *Haemophilus* sp. HMSC71H05, and *H. parainfluenzae* (Figure 1B; Data S1B)— which are usually more associated with oral microbiomes[29] and gastrointestinal problems[30]—and *Odoribacter splanchnicus* and *Veillonella* sp. T110116, which instead are gut commensals exerting potential beneficial effects.[31,32] While there is thus a great degree of overlap between Ethiopian and Ghanaian and Tanzanian microbiomes, remarkable attributes in microbiome compositions and structures still differentially characterize each specific (non-westernized) community.

Previously uncharacterized species strengthen the similarity between Ethiopian and non-westernized infant microbiomes

Microbiome members of individuals from non-westernized populations are particularly underrepresented by current cultivation-based genomic catalogs causing metagenomic profiling approaches to miss large fractions of the microbial diversity in the samples. Recent efforts showed that metagenomic assembly can partially mitigate this problem,[23,25,33,34] but also metagenomic surveys are deeply biased toward the microbiome of westernized populations.[35,36] To have a more thorough overview of the microbiome composition of the Ethiopian cohort as well as of the cohort we used for contextualizing them, we applied *de novo* metagenomic assembly and binning on all the 702 metagenomes considered here to identify also previously uncharacterized species (see STAR Methods).

From the Ethiopian cohort, we reconstructed 1,134 metagenome-assembled genomes (MAGs, considered of sufficient quality with completeness >50% and contamination <5%; see STAR Methods) that were assigned to 376 species-level genome bins (SGBs, spanning 5% genetic diversity; see STAR Methods). Among all SGBs, 116 were classified as “known” (kSGBs) as they included reference genomes available in public databases and 260 as “unknown” (uSGBs, comprising 751 MAGs) lacking reference genomes (Data S2A). As a proxy for species-sharing across cohorts, we then surveyed the fraction of SGBs that were found both in the Ethiopian and westernized and/or non-westernized populations. Of all the 376 SGBs, 128 were uniquely found in the Ethiopian cohort (totaling 231 MAGs) and not in the other westernized and non-westernized cohorts considered in this study. The Ethiopian cohort instead shared 51.4% of the SGBs (n = 18, 3 kSGBs and 15 uSGBs, representing 98 Ethiopian MAGs, 8.64% of the total) found in both Ghanaian and Tanzanian samples but not in westernized samples, and 12.2% (n = 30, 21 kSGBs and 7 uSGBs, representing 71 Ethiopian MAGs, 6.26% of the total) of the 245 SGBs found in both Italian and Swedish cohorts but not in Ghana and Tanzania. This SGB-based analysis showed a degree of microbial overlap between the considered non-westernized populations that is larger than with the westernized ones despite the lower number of samples and SGBs available for them (Data S2B). With the aim of identifying SGBs shared across populations despite differences in lifestyle and geographic location, we found a “core” of 50 SGBs (31 kSGBs and 19 uSGBs) present at least once in all five cohorts (representing 25.6% ± 2.7% of the MAGs of each single cohort). Nine of them (7 kSGBs and 2 uSGBs) also had an overall mean prevalence >10% in the 700 samples considered in this study (Data S2B), thus potentially representing SGBs tightly linked with the human host irrespectively of lifestyle and geography (i.e., universal environment-independent microbiome features). Among them, *Eubacterium rectale* SGB4933 (36 MAGs in Ethiopian cohort) and *Prevotella copri* Clade A SGB1626 (15 MAGs[26]) were the most prevalent kSGBs in the Ethiopian cohort (72% and 30%, respectively).

E. rectale SGB4933, *Ruminococcus bromii* SGB4285, *Faecalibacterium prausnitzii* SGB15316, and *Firmicutes bacterium* AF22 6AC SGB4910 were also part of the 18 SGBs (1 uSGB) consistently detected in infants of all cohorts (Figure 1C; DataS2C). Phylogenetic strain-level profiling of these common infant SGBs revealed that for some of them, strains from non-westernized infants tended to cluster into specific clades (Figure 1D).[23,37–39] This was particularly clear for *R. bromii* SGB4285, *F. prausnitzii* SGBs 15316 and 15318, and *E. rectale* SGB4933 and the related *Eubacterium* uSGB4290 (Figures 1D and S1C) but not for two of the most prevalent SGBs in infants, namely *Parabacteroides distasonis* SGB1934 and *Phocaeicola vulgatus* SGB1814, which showed no specific lifestyle-dependent strain similarity pattern (Figure S1C).

For the infant microbiome, 79 SGBs (of which 62 are uSGBs) were uniquely found in Ethiopian infants and never observed in infants of other cohorts (Figure 1C). Interestingly, 19 SGBs (14 uSGBs) were found in all the considered non-westernized infant cohorts but were never present in the westernized ones (Figure 1C). Of these, 10 SGBs were assigned to the *Prevotellaceae* family and 5 were unassigned at the phylum level (Data S2C), suggesting that not only non-westernized adults[22,24,40] but also infants, which are still poorly sampled in studies focusing on non-westernized communities, harbor a large amount of uncharacterized microbial diversity in their gut. This microbial under-characterization in non-westernized populations was confirmed by the detection of 90 uSGBs that were uniquely found in non-westernized and Ethiopian infants’ metagenomes (in comparison, only 24 SGBs were found in both Ethiopian and westernized infants but not in non-westernized ones; Figure 1C).

Ethiopian infants share a lower fraction of the microbiome with their mothers than in westernized populations

We then evaluated to which extent the infant microbiome composition could be explained by the extent of microbes that are shared with the mother,[10,15–17,41] a phenomenon that has never been described at the strain level in non-westernized populations and that might in part be responsible for the differences observed later in life between microbiomes of populations with westernized

CellPress
OPEN ACCESS

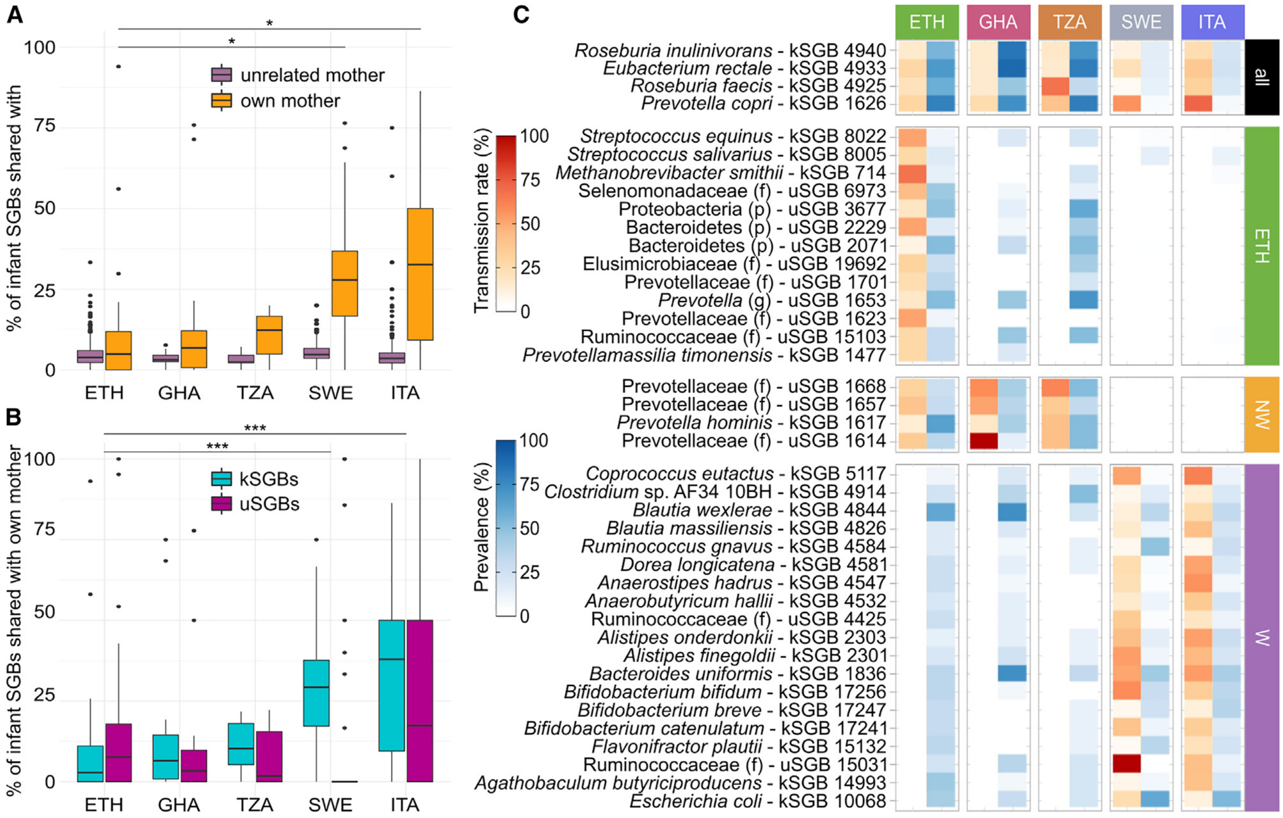

Figure 2. Ethiopian infants and their mothers share fewer and uncharacterized microbiome species

(A) Percentage of infant microbiome species that are also shared at the strain level with their own mother (yellow) or with unrelated mothers from the same cohort (pink) in different countries. With respect to westernized infants, Ethiopian infants tend to share a more limited fraction of the intestinal species with their mothers, in concordance with the other two non-westernized cohorts. This could be due to a greater contribution of other sources, such as other family members or the environment (*p value < 0.05). The box plots show the first and third quartiles (boxes) and the median (middle line); the whiskers extend up to 1.5× the IQR.

(B) Percentage of infant kSGBs (light blue) and uSGBs (purple) that are also shared at the strain level with their own mother. Ethiopian infants share a larger fraction of uSGBs than kSGBs with their mothers, contrary to infants from other countries, especially westernized ones (***p value < 0.001). The box plots show the first and third quartiles (boxes) and the median (middle line); the whiskers extend up to 1.5× the IQR.

(C) SGBs shared at the strain level between infant and mother in all cohorts or in the Ethiopian, non-westernized (including Ethiopian), or westernized cohorts only. For each SGB, strain-sharing rate (red scale) is reported as the percentage of the strains found in the infant that is also found in the corresponding mother. SGB prevalence in infants of each country is also reported (blue scale). The taxonomic label reported for uSGBs specifies if the assignment is at the genus (g), family (f), or phylum (p) level.

See also Figure S2 and Data S2.

and non-westernized lifestyles. To do this, we extended our previous methodology[16] by applying MetaPhlAn 3 and StrainPhlAn 3[28] on a collection of SGB-specific marker genes, enabling the reconstruction of strain-level phylogenies for SGBs of interest (including uSGBs; see STAR Methods). For each mother-infant pair, this approach allowed us to verify whether the strains of an SGB found in both microbiomes are the same strain—thus inferring possible mother-to-infant transmission—or distinct strains and thus likely acquired independently.

We found that Ethiopian infants shared with their mothers a median of 4.9% of the SGBs found in their microbiome at the stain level, in line with other non-westernized cohorts (median 7.6%) but substantially lower than westernized infants (median 28.6%, Fisher test p value < 2.2E−16; Figure 2A). Of note, Ethiopian and non-westernized infants had on average 35.6 SGBs that could be profiled at the strain level, which is higher than that for westernized infants (24.3 SGBs), suggesting the lower fraction of microbes shared in non-westernized microbiomes is not due to technical artifacts and may be in part explained by a much higher microbiome richness, possibly due to increased acquisition of microbiome members from other environmental or family sources.[42,43] Similarly, the presence of C-section samples in the Italian and Swedish cohorts (15/156 and 30/300, respectively) showed no effect on the strain-sharing comparison by lifestyle. For instance, both the Italian and Swedish cohorts had a higher strain-sharing rate than the Ethiopian, Ghanaian, and Tanzanian populations, which is the opposite effect exerted by C-section samples in the Swedish cohort (i.e., reduction of the strain-sharing rate; Figure S2A).

Most of the strains shared between Ethiopian mothers and their infants are from uncharacterized species

In the Ethiopian cohort, the taxa from which mother-infant strain sharing was inferred belonged to uncharacterized SGBs (i.e.,

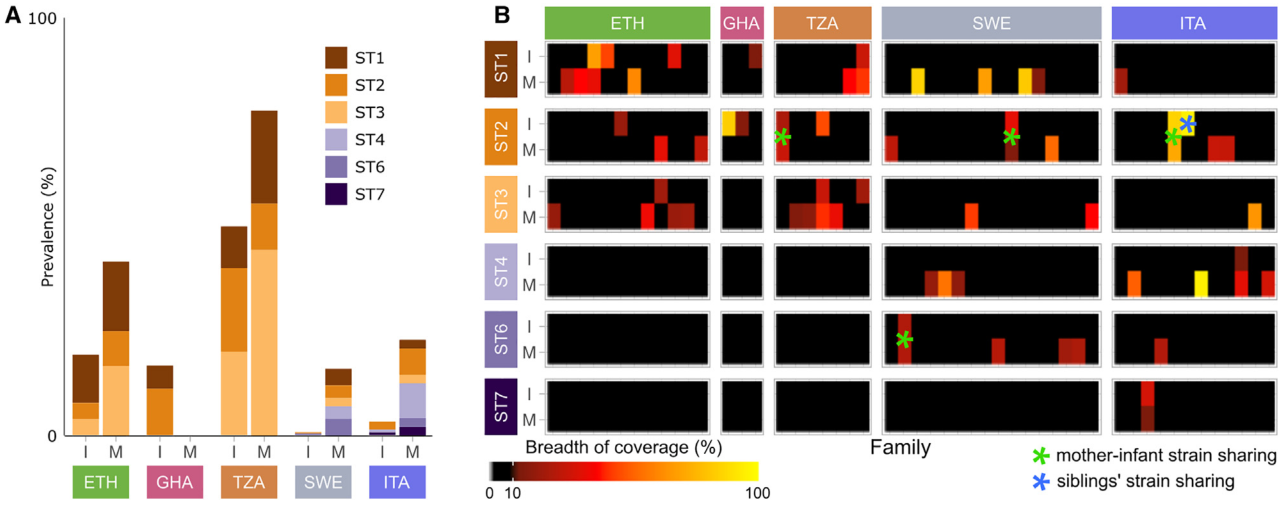

Figure 3. *Blastocystis* species are rarely shared at the strain level between mothers and their infants

(A) *Blastocystis* sp. subtypes (STs) prevalence in the different cohorts of infants (I) and mothers (M). *Blastocystis* spp. prevalence is much higher in Ethiopian and non-westernized infant metagenomes than in westernized ones, even though only three STs are found in the former. Westernized gut metagenomes generally show a lower prevalence of *Blastocystis* spp. but contain up to six different STs. Further details available in Table S2.

(B) Breadth of coverage and strain sharing (*) for different *Blastocystis* sp. STs in different countries. Samples reported here are those for which either the mother or the infant (or both) tested positive (BoC > 10%; see STAR Methods) for at least one ST. ST2 was shared at the strain level in all cases where both the infant (I) and the mother (M) were positive for that specific ST. ST2 was also shared between two siblings (blue *). For more details, see Figure S2B.

uSGBs; Figure 2B), in stark contrast with what was observed in the Swedish cohort, where infants shared an extremely limited fraction of uSGs with their own mothers (Figure 2B). Indeed, of the 13 SGBs that were specifically shared only in the Ethiopian cohort, only 4 were kSGBs, whereas the other 9 were uSGBs taxonomically unassigned at the species (n = 1), genus (n = 5), and class (n = 3) levels (Figure 2C). Among these, the *Selenomonadaceae* uSGB6973 was not only highly prevalent in Ethiopian mother-infant pairs (42.9%) but also highly shared (17.9% of positive pairs), while being present only in one individual and never shared in both Ghana and Tanzania (Figure 2C). Similarly, all the four SGBs (3 uSGBs) that were only shared in the Ethiopian and in the other non-westernized cohorts belonged to the *Prevotellaceae* family, and they were present on average in 37.7% and shared in 14.3% of the non-westernized mother-infant pairs while completely absent in Italian and Swedish ones (Figure 2C). On the contrary, there are also 19 SGBs (2 uSGBs) that are inferred to be shared only in these two European cohorts. These include *Escherichia coli* kSGB10068 and four *Bifidobacterium* species (*B. uniformis*, *B. bifidum*, *B. breve*, and *B. catenulatum*) that have been found to be highly transmitted from mother to child in several studies[10,15–17,41] and that are also present but not shared in pairs from non-westernized populations (Figure 2C). Although we also detected a small set of kSGBs that were consistently shared between mother and infant in all five cohorts (*Prevotella copri* Clade A SGB1626, *Eubacterium rectale* SGB4933, *Roseburia inulinivorans* SGB4940, and *Roseburia faecis* SGB4925; Figure 2C), these results further highlight how differences in lifestyle may impact not only the composition of the microbiome but also the set of microbes that are acquired from the mother during the first years of life.

Blastocystis sp. is present in both mothers and infants of all cohorts but is only rarely shared

Given that mother-to-infant transmission may not be limited to the prokaryotic fraction of the microbiome, we investigated the strain-sharing pattern of some eukaryotes that are known to be present in the human gut[44–46] (Table S1). While intestinal parasites such as *Cryptosporidium*, *Entamoeba*, and *Giardia* spp. were never or rarely identified and at very low relative abundance in our samples (*Cryptosporidium* and *Entamoeba* spp. prevalence = 0/700, *Giardia* spp. prevalence = 4/700; Table S1), we identified *Blastocystis* sp. in 10 Ethiopian mothers and 5 Ethiopian infants (Table S2) by applying a previously validated pipeline.[47] *Blastocystis* sp. was detected in 30% of the samples with three distinct sequence types (STs), namely ST1, ST2, and ST3. In concordance with previous findings,[48] we confirmed that *Blastocystis* sp. was never detected in Ethiopian newborns (the youngest positive child was 21 months old), suggesting that this eukaryote is acquired later in life also in non-westernized communities where its prevalence is higher in the adults. For 3 of the 5 colonized Ethiopian infants, we found that the mother was colonized as well but in only one case with the same ST (namely ST1 in family D07; Figure 3B; Table S2).

We then extended our analysis also to the other mother-infant cohorts and found that in the non-westernized populations *Blastocystis* sp. was present at a similar average prevalence (37%) and with the same three STs (i.e., ST1, ST2, and ST3; Figure 3A). On the contrary, in the westernized cohorts, *Blastocystis* sp. was present in only 8% of the samples and with three additional STs (i.e., ST4, ST6, and ST7; Figure 3A). Only one Italian newborn was found positive for *Blastocystis* sp. on the day of delivery but not on the following days (Table S2), suggesting potential transmission that did not lead to stable colonization. Overall,

Table 1. Species found in injera samples (E1 and E2) and their prevalence in Ethiopian (ETH), non-westernized (NW), and westernized (W) gut metagenomes.

	Rel. ab. in injera (%)		Prevalence (%)			
Species	E1	E2	ETH	NW	W	p value
Lacticaseibacillus paracasei SGB7142	56.06	48.56	4	0	8.1	n.s.
Lactiplantibacillus xiangfangensis SGB7204	36.62	32.90	2	0	0	1.04E−03
Gluconobacter oxydans SGB1165	1.15	9.52	0	0	0	–
Fructilactobacillus sanfranciscensis SGB7164	4.71	2.10	28	0	0.2	4.68E−36
Gluconobacter cerinus SGB1160	0.60	3.60	0	0	0	–
Gluconobacter japonicus SGB1161	0.44	2.42	0	0	0	–
Acetobacter orientalis SGB1187	0.14	0.67	0	0	0	–
Latilactobacillus curvatus SGB7249	0.17	0.10	0	0	0.3	n.s.
Secundilactobacillus silagei SGB7208	0.05	0.03	0	0	0	–
Pediococcus parvulus SGB7180	0.05	0.02	46	0	0	1.05E−63

Only species with a relative abundance (rel. ab.) > 0.05% in at least one of the two injera samples are reported. Mann-Whitney U test FDR-corrected p values highlight injera species that are statistically more prevalent in Ethiopian gut metagenomes than in westernized ones. n.s. = not significant. See also Tables S3 and S4.

westernized infants had a much lower prevalence of *Blastocystis* spp. (3%) with a median age of 4 years and 7 months for the positive individuals. Non-westernized infants, including Ethiopian ones, had instead a higher prevalence (29%) but a very similar median age (6 years), which again suggests that acquisition of *Blastocystis* sp. occurs later in life independently of lifestyle. In cases in which both the mother and the infant were colonized by *Blastocystis* spp., infants of non-westernized cohorts shared the same ST with their mothers only in 3 out of 5 cases (Figure 3B), as already observed in the Ethiopian cohort, with siblings belonging to the same family carrying different STs. On the contrary, all mothers of positive westernized offspring were carrying the same ST as their children (Figure 3B), suggesting that in non-westernized cohorts the environmental acquisition might be more relevant than in westernized cohorts.

To test whether *Blastocystis* sp. was shared at the strain level in the mother-infant pairs sharing the same ST, we performed a single-nucleotide polymorphism (SNP)-calling analysis (see STAR Methods). Among all considered STs, only ST2 and ST6 showed the presence of the same strain in the mother and her infant (Figures 3B and S2B). ST2 was also the only ST shared at the strain level in all cases in which it was present both in the mother and the infant, and also in one case of two siblings sharing the same ST (Figure S2B). This was true across 3 different cohorts, suggesting that ST2 might be particularly prone to be shared between mother and infant in the first years of life.

Impact of a traditional fermented teff-based food product as a source of specific gut microbiome members

Teff (*Eragrostis tef*) is a cereal typically grown in the tropics and especially in Ethiopia, where it represents a major food grain. Teff is commonly used in Ethiopia to make injera, a typical fermented bread-like food, but also other fermented products, such as soups and alcoholic beverages.[49] Teff is traditionally naturally fermented by saving part of the liquid that forms at the top of the fermentation vessel (ersho) to start a new fermentation process driven by both prokaryotic and eukaryotic microbes.[50–52]

To better understand the role of so widely used fermented teff products in the dynamics of the gut microbiome of Ethiopian mothers and infants and mother-to-infant microbiome strain sharing, we collected two samples of fermented dough and shotgun sequenced their microbial communities. *Lacticaseibacillus paracasei* subsp. *paracasei* SGB7142 (previously *Lactobacillus paracasei*) and *Lactiplantibacillus xiangfangensis* SGB7204 (previously *Lactobacillus xiangfangensis*) were the most abundant microbes in both samples, where they represented 92.7% and 81.5% of the microbial community (Table 1). Only eight other species were present at a relative abundance >0.05% in at least one of the two samples (Table 1). These included three previously *Lactobacillus* species (*Fructilactobacillus sanfranciscensis*, *Latilactobacillus curvatus*, and *Secundilactobacillus silagei*), three *Gluconobacter* species (*G. oxydans*, *G. cerinus*, and *G. japonicus*), *Acetobacter orientalis*, and *Pediococcus parvulus*. While lactic acid bacteria (LAB) belonging to the former genus *Lactobacillus* and genus *Pediococcus* are commonly found in different teff sourdoughs,[50,51] the acetic acid bacteria (AAB) *Gluconobacter* and *Acetobacter* spp. were not described in fermented teff products before, even if they are commonly found in other fermented foods, such as kombucha and kefir, but also in the cocoa bean and buckwheat fermentation, and the sourdough fermentation starter jiaozi.[53–55]

Given that teff-based porridges and gruels (i.e., genfo) are traditionally eaten by new mothers and weaning infants, we investigated whether microbes found in the teff dough were also present in the gut metagenomes of the Ethiopian community. Only four out of the ten species found in the dough were also present in Ethiopian individuals (i.e., three previously *Lactobacillus*species and *Pediococcus parvulus*; Table 1), although their relative abundance was always <0.05%. Three dough species were statistically more prevalent in Ethiopian than in westernized and other non-westernized metagenomes (Mann-Whitney U test, FDR-corrected p values < 0.05; Table 1): *Fructilactobacillus sanfranciscensis* (previously *Lactobacillus sanfranciscensis*), *P. parvulus*, and *L. xiangfangensis*. Strain-level analysis was further conducted on these species to test the hypothesis that fermented teff products could be

valuable sources of these unusual members of the gut microbiome.

For *F. sanfranciscensis* (found in 14 gut microbiomes from Ethiopia and in only 1 from westernized cohorts, p value 4.68E−36; Table 1), gut metagenomes' reads were mapped to the MAGs reconstructed from the dough and other available reference genomes (see STAR Methods). The dough MAGs exhibited the highest breadth of coverage (thus likely best match) for 50% of the Ethiopian samples, whereas the remaining ones mapped better against other reference genomes (i.e., GCA_009496975 in 6 cases and GCA_000225325 in 1 case; Table S3). However, also when these other genomes were the best match for the Ethiopian strains, the dough MAGs were on median the second and fourth closest references and only the 21st and 24th closest to the only strain of *F. sanfranciscensis* from westernized populations (Table S3). Additionally, all top alignments with the dough MAGs for *F. sanfranciscensis* were the Ethiopian strains (Table S3). This provides strong evidence for teff fermented products to be a nutritional source of *F. sanfranciscensis*, a species otherwise very rarely found in gut metagenomes of westernized and non-westernized communities.[56] We could not perform the same kind of analysis for the other two dough species. Despite its high prevalence in Ethiopian samples (46%), *P. parvulus* was indeed detected only at a 0.03% mean relative abundance in the dough, therefore making it not feasible to reconstruct a MAG to be used as a reference for mapping (Table 1). On the contrary, MAGs of *L. xiangfangensis* were reconstructed from dough, but this species was detected only in Ethiopian samples, therefore a comparison with external cohorts was not possible. Our analysis suggests that locally produced fermented teff products could be the source of these two unusual species that were detected in the Ethiopian cohort but never found in the set of 650 metagenomes not belonging to the Ethiopian community (Table 1).

DISCUSSION

In this study, we assessed the strain-sharing patterns of gut microbiome members in a cohort of Ethiopian mothers and infants[26] in the context of other westernized and non-westernized cohorts from Ghana,[26] Tanzania,[26] Sweden,[10] and Italy,[15,16] which we expanded with 70 newly sequenced stool metagenomes from Italian infants and their mothers (total analyzed fecal metagenomes = 700). This analysis highlighted that Ethiopian and other children with non-westernized lifestyles had a more similar microbiome composition that was instead different from the one of children with westernized lifestyles. Similar, albeit less marked, patterns were already observed for adults[22–25] with different lifestyles but were never investigated in younger individuals. Newborns under 1 year of age indeed showed a higher degree of microbiome composition similarity regardless of their lifestyle (westernized or non-westernized), probably driven by their milk-based diet. Infants from the Ethiopian and other non-westernized populations also shared a smaller fraction of the strains in their gut microbiome with their mothers, when compared with westernized ones, with a large fraction of the shared strains belonging to uncharacterized species. Among these, we identified some species that are highly prevalent and shared between mother and infant only in the Ethiopian population, suggesting potential population effects driven by geographically confined differences. An example is the locally produced teff-based fermented sourdough that we newly sequenced in this study and that proved to be a source of microbial strains uniquely present in the Ethiopian cohort, albeit probably only transiently.

Most of the studies about infant microbial colonization have been conducted in westernized populations, and thus the effect of different lifestyles on bacterial seeding has been largely underexplored. Previous studies in adults have described differential microbiota patterns in non-westernized populations, highlighting, for example, *Prevotella* spp. as a marker for non-westernized microbiomes.[26,57] Similarly to what was described in adults, we found an enrichment in *Prevotella* spp. such as *P. copri*, *P. stercorea*, and *Prevotella* sp. AM42 24 in Ethiopian infants' microbiomes, when compared with infants from westernized communities. Recently, a study based on 16S rRNA gene sequencing confirmed these results in children from a non-westernized Gambian community, where *P. copri*, *F. prausnitzii*, and *P. stercorea* were the most abundant species in the infant gut microbiome,[58] and they increased gradually with age. Interestingly, we also reported that the differences between Ethiopian and westernized children increased with age, suggesting a crucial role of the environment and diet in the microbiome composition divergence between lifestyles.

Here, we started investigating the extent to which mother-infant strain-sharing affects the observed microbiome differences in different populations with divergent lifestyles. Our results revealed a lower strain sharing in the Ethiopian mother-infant pairs and other non-westernized cohorts than in the westernized ones, and also differences in the shared species. While *Escherichia coli* and *Bifidobacterium* spp. are the most shared within the European pairs, as previously described,[10,15–17,41] uncharacterized species, mainly from *Selenomonadaceae* and *Prevotellaceae* families, are among the most shared with the mother during the first years of life in non-westernized populations. To our knowledge, this is the first study addressing mother-infant strain sharing in non-westernized communities and reporting how environmental conditions affect not only infant microbiome composition but also its acquisition. Indeed, even though diet could be a determinant in the shaping of the infant microbiome, our results about the mother-infant sharing of mainly unassigned *Prevotellaceae* spp. could not discard that maternal seeding during early life may also contribute to the divergence in microbiota composition associated with non-westernized communities. In fact, the higher presence of unknown bacterial species not present in the reference databases is also a feature of the non-westernized communities,[23] and our results highlight that these previously uncharacterized species are also shared at the strain level between mother and infant. Further studies are needed to decipher the potential impact of these strains shared with the mother on infant colonization and development in the long term and to elucidate which lifestyle factors impact transmission, which we could not assess in the present work.

As the microbiome is not composed only of prokaryotic organisms, we also explored the potential transmission of the less investigated eukaryotic fraction of the infant microbiome. *Blastocystis* sp., which was the most prevalent (30%) eukaryote in the non-westernized infant microbiome, was detected only in children older than 1 year, supporting the hypothesis that it would

be acquired later in life. In agreement with other studies,[48,59,60] we found a higher presence of this eukaryotic group in non-westernized cohorts compared with westernized communities. Cinek et al. reported that the prevalence and subtypes of *Blastocystis* varied among six different geographical locations and it would be also related to the bacterial fraction of the infant microbiome.[61] Thus, we investigated whether this difference could be partially explained by strain sharing with their mothers. However, our results suggest that the mother-infant sharing of *Blastocystis* spp. would be relevant in the case of westernized countries but much less in non-westernized communities where the environment may be the main source for these species. Overall, our observations highlight the environment (likely as a medium from fecal sources) as one of the main microbial sources in non-westernized countries, showing that maternal seeding may play a more limited role in both prokaryotic and eukaryotic seeding of the infant's gut.

Due to the large impact of the environment on the infant microbiome composition, we further hypothesized that diet, and specifically traditional fermented food due to its capacity to modulate gut microbiota,[62,63] could also contribute to the increased microbial diversity of Ethiopian infants metagenomes with transiently present species. To test this, we included in the metagenomic analysis two samples of injera, a highly consumed food made of teff sourdough fermented for 24–96 h, depending on the optimum temperature, which contain higher counts of LAB and yeast (LAB to yeast ratio of 10:1 to 100:1).[64] As was expected, we found a higher presence of LAB, mainly belonging to the former genus *Lactobacillus* and to *Pediococcus* species, in the injera but also AAB which are commonly described in other fermented products. Indeed, we also found these species with a higher prevalence in the Ethiopian cohort compared with the rest of the populations, including other non-westernized ones, where these uncommon taxa are barely detected. Despite the limitations in genome reconstruction from injera metagenomes derived from the low relative abundance of these species, our results suggest that the highly consumed fermented food would be a source of LAB for the gut microbiome, contributing to the difference in the microbial patterns observed in this population. However, the viability of these species needs further investigation, as it is possibly a transient presence in the gut due to continued consumption of injera in the Ethiopian population. These analyses are limited by the small sample size and by the lack of metagenomes of fermented food items consumed by new mothers and weaning infants from the other surveyed westernized and non-westernized cohorts, and further studies are needed to understand the role of fermented food in the initial phases of microbiome establishment.

Overall, the work presented here highlights how lifestyle can impact microbiome composition not only through the different food sources, drug availability, and environmental factors but potentially also through different patterns of mother-to-infant sharing of microbiome strains that strongly differ between westernized and non-westernized lifestyles. These preliminary findings on mother-infant strain sharing strongly support the need for a more comprehensive understanding of maternal transmission in light of geography and lifestyle, which can be achieved by diversifying sampling cohorts according to geography and lifestyle and intensifying research efforts on maternal-infant gut microbiome transmission.

STAR★METHODS

Detailed methods are provided in the online version of this paper and include the following:

- KEY RESOURCES TABLE
- RESOURCE AVAILABILITY
  - Lead contact
  - Materials availability
  - Data and code availability
- EXPERIMENTAL MODEL AND SUBJECT DETAILS
  - Mother-infant cohorts surveyed in this study
  - Lifestyle of the Ethiopian communities assessed in this study
  - Definition of Westernized and Non-Westernized lifestyles
- METHOD DETAILS
  - Newly sequenced metagenomes
  - MAGs reconstruction, clustering, and taxonomic assignment
  - Species-level and strain-level profiling of mother-infant cohorts
  - Phylogenetic analysis of the SGBs shared across all infants cohorts
  - *Blastocystis* sp. profiling and SNP calling
  - *Fructilactobacillus sanfranciscensis* (previously *Lactobacillus sanfranciscensis*) mapping
- QUANTIFICATION AND STATISTICAL ANALYSIS

SUPPLEMENTAL INFORMATION

Supplemental information can be found online at https://doi.org/10.1016/j.cub.2023.04.011.

ACKNOWLEDGMENTS

This work was supported by the European Research Council (ERC-STG project MetaPG-716575) to N.S., by MIUR "Futuro in Ricerca" (grant no. RBFR13EWWI_001) to N.S., by the European H2020 program (ONCOBIOME-825410 project and MASTER-818368 project) to N.S., by the National Cancer Institute of the National Institutes of Health (1U01CA230551) to N.S., by the Premio Internazionale Lombardia e Ricerca 2019 to N.S., by the Italian Ministry of Health with Ricerca Corrente and 5x1000 funds, and by the EMBO ALTF 593-2020 to M.V.-C. The authors would like to acknowledge support from the European Research Council (ERC-STG project MAMI, ref. 639226) and PROMETEO GVA (ref. 012/2020) to M.C.C. and M.S.-R., respectively, and from Plan GenT project (CDEIGENT 2020) to R.C.-R. Finally, M.C.C., R.C.-R., and M.S.-R. acknowledge the award of the Spanish government MCIN/AEI to the IATA-CSIC as Center of Excellence Accreditation Severo Ochoa (CEX2021-001189-S/MCIN/AEI/10.13039/501100011033).This article is part of a project that has received funding from the European Union's Horizon 2020 Research and Innovation Programme (GA no. 949742 ERC-HealthXCross) to R.R.

We would like to thank all the families from Gimbichu and Igúkura who were involved in the study research; the San Pablo Apóstol Missionary Community (MCSPA); and also the MOSSolidaria ONGD and the GeNaPi Project Team, including personnel from the UV and CSIC.

AUTHOR CONTRIBUTIONS

Conceptualization, S.M., E.P., M.C.C., and N.S.; software, K.D.H., F. Asnicar, A.B.-M., F.C., P.M., and E.P.; formal analysis, S.M., K.D.H., F. Asnicar, F.C., D.G., and E.P.; investigation, S.M., F. Armanini, and E.P.; resources, L.A., M.V.C., M.P., M.O., M.C.C., and N.S.; data curation, S.M., M.S.-R., F.

Armanini, and M.C.C.; writing—original draft, S.M., M.S.-R., and E.P.; writing—review and editing, S.M., M.S.-R., F. Asnicar, P.M., F.P., M.V.-C., R.R., R.C.-R., E.P., M.C.C., and N.S.; visualization, S.M.; supervision, N.S.; project administration, S.M., M.C.C., and N.S.; funding acquisition, M.C.C. and N.S.

DECLARATION OF INTERESTS

The authors declare no competing interests.

Received: June 17, 2022
Revised: February 28, 2023
Accepted: April 5, 2023
Published: April 27, 2023

REFERENCES

1. Dominguez-Bello, M.G., Costello, E.K., Contreras, M., Magris, M., Hidalgo, G., Fierer, N., and Knight, R. (2010). Delivery mode shapes the acquisition and structure of the initial microbiota across multiple body habitats in newborns. Proc. Natl. Acad. Sci. USA *107*, 11971–11975.
2. Dominguez-Bello, M.G., De Jesus-Laboy, K.M., Shen, N., Cox, L.M., Amir, A., Gonzalez, A., Bokulich, N.A., Song, S.J., Hoashi, M., Rivera-Vinas, J.I., et al. (2016). Partial restoration of the microbiota of cesarean-born infants via vaginal microbial transfer. Nat. Med. *22*, 250–253.
3. Bokulich, N.A., Chung, J., Battaglia, T., Henderson, N., Jay, M., Li, H., Lieber, A.D., Wu, F., Perez-Perez, G.I., Chen, Y., et al. (2016). Antibiotics, birth mode, and diet shape microbiome maturation during early life. Sci. Transl. Med. *8*, 343ra82.
4. Chu, D.M., Ma, J., Prince, A.L., Antony, K.M., Seferovic, M.D., and Aagaard, K.M. (2017). Maturation of the infant microbiome community structure and function across multiple body sites and in relation to mode of delivery. Nat. Med. *23*, 314–326.
5. Shao, Y., Forster, S.C., Tsaliki, E., Vervier, K., Strang, A., Simpson, N., Kumar, N., Stares, M.D., Rodger, A., Brocklehurst, P., et al. (2019). Stunted microbiota and opportunistic pathogen colonization in caesarean-section birth. Nature *574*, 117–121.
6. Yassour, M., Vatanen, T., Siljander, H., Hämäläinen, A.M., Härkönen, T., Ryhänen, S.J., Franzosa, E.A., Vlamakis, H., Huttenhower, C., Gevers, D., et al. (2016). Natural history of the infant gut microbiome and impact of antibiotic treatment on bacterial strain diversity and stability. Sci. Transl. Med. *8*, 343ra81.
7. Stearns, J.C., Simioni, J., Gunn, E., McDonald, H., Holloway, A.C., Thabane, L., Mousseau, A., Schertzer, J.D., Ratcliffe, E.M., Rossi, L., et al. (2017). Intrapartum antibiotics for GBS prophylaxis alter colonization patterns in the early infant gut microbiome of low risk infants. Sci. Rep. *7*, 16527.
8. Vatanen, T., Kostic, A.D., d'Hennezel, E., Siljander, H., Franzosa, E.A., Yassour, M., Kolde, R., Vlamakis, H., Arthur, T.D., Hämäläinen, A.-M., et al. (2016). Variation in microbiome LPS immunogenicity contributes to autoimmunity in humans. Cell *165*, 842–853.
9. Uzan-Yulzari, A., Turta, O., Belogolovski, A., Ziv, O., Kunz, C., Perschbacher, S., Neuman, H., Pasolli, E., Oz, A., Ben-Amram, H., et al. (2021). Neonatal antibiotic exposure impairs child growth during the first six years of life by perturbing intestinal microbial colonization. Nat. Commun. *12*, 443.
10. Bäckhed, F., Roswall, J., Peng, Y., Feng, Q., Jia, H., Kovatcheva-Datchary, P., Li, Y., Xia, Y., Xie, H., Zhong, H., et al. (2015). Dynamics and stabilization of the human gut microbiome during the first year of life. Cell Host Microbe *17*, 852.
11. O'Sullivan, A., Farver, M., and Smilowitz, J.T. (2015). The influence of early infant-feeding practices on the intestinal microbiome and body composition in infants. Nutr. Metab. Insights *8* (*Suppl 1*), 1–9.
12. Davis, E.C., Wang, M., and Donovan, S.M. (2017). The role of early life nutrition in the establishment of gastrointestinal microbial composition and function. Gut Microbes *8*, 143–171.
13. Timmerman, H.M., Rutten, N.B.M.M., Boekhorst, J., Saulnier, D.M., Kortman, G.A.M., Contractor, N., Kullen, M., Floris, E., Harmsen, H.J.M., Vlieger, A.M., et al. (2017). Intestinal colonisation patterns in breastfed and formula-fed infants during the first 12 weeks of life reveal sequential microbiota signatures. Sci. Rep. *7*, 8327.
14. Fallani, M., Amarri, S., Uusijarvi, A., Adam, R., Khanna, S., Aguilera, M., Gil, A., Vieites, J.M., Norin, E., Young, D., et al. (2011). Determinants of the human infant intestinal microbiota after the introduction of first complementary foods in infant samples from five European centres. Microbiology (Reading) *157*, 1385–1392.
15. Asnicar, F., Manara, S., Zolfo, M., Truong, D.T., Scholz, M., Armanini, F., Ferretti, P., Gorfer, V., Pedrotti, A., Tett, A., and Segata, N. (2017). Studying vertical microbiome transmission from mothers to infants by strain-level metagenomic profiling. mSystems *2*, https://doi.org/10.1128/mSystems.00164-16.
16. Ferretti, P., Pasolli, E., Tett, A., Asnicar, F., Gorfer, V., Fedi, S., Armanini, F., Truong, D.T., Manara, S., Zolfo, M., et al. (2018). Mother-to-infant microbial transmission from different body sites shapes the developing infant gut microbiome. Cell Host Microbe *24*. 133.e5–145.e5.
17. Yassour, M., Jason, E., Hogstrom, L.J., Arthur, T.D., Tripathi, S., Siljander, H., Selvenius, J., Oikarinen, S., Hyöty, H., Virtanen, S.M., et al. (2018). Strain-level analysis of mother-to-child bacterial transmission during the first few months of life. Cell Host Microbe *24*. 146.e4–154.e4.
18. David, L.A., Maurice, C.F., Carmody, R.N., Gootenberg, D.B., Button, J.E., Wolfe, B.E., Ling, A.V., Devlin, A.S., Varma, Y., Fischbach, M.A., et al. (2014). Diet rapidly and reproducibly alters the human gut microbiome. Nature *505*, 559–563.
19. Valdes, A.M., Walter, J., Segal, E., and Spector, T.D. (2018). Role of the gut microbiota in nutrition and health. BMJ *361*, k2179.
20. Asnicar, F., Berry, S.E., Valdes, A.M., Nguyen, L.H., Piccinno, G., Drew, D.A., Leeming, E., Gibson, R., Le Roy, C., Khatib, H.A., et al. (2021). Microbiome connections with host metabolism and habitual diet from 1,098 deeply phenotyped individuals. Nat. Med. *27*, 321–332.
21. Schwartz, D.J., Langdon, A.E., and Dantas, G. (2020). Understanding the impact of antibiotic perturbation on the human microbiome. Genome Med. *12*, 82.
22. Yatsunenko, T., Rey, F.E., Manary, M.J., Trehan, I., Dominguez-Bello, M.G., Contreras, M., Magris, M., Hidalgo, G., Baldassano, R.N., Anokhin, A.P., et al. (2012). Human gut microbiome viewed across age and geography. Nature *486*, 222–227.
23. Pasolli, E., Asnicar, F., Manara, S., Zolfo, M., Karcher, N., Armanini, F., Beghini, F., Manghi, P., Tett, A., Ghensi, P., et al. (2019). Extensive unexplored human microbiome diversity revealed by over 150,000 genomes from metagenomes spanning age, geography, and lifestyle. Cell *176*. 649.e20–662.e20.
24. De Filippo, C., Cavalieri, D., Di Paola, M., Ramazzotti, M., Poullet, J.B., Massart, S., Collini, S., Pieraccini, G., and Lionetti, P. (2010). Impact of diet in shaping gut microbiota revealed by a comparative study in children from Europe and rural Africa. Proc. Natl. Acad. Sci. USA *107*, 14691–14696.
25. Nayfach, S., Shi, Z.J., Seshadri, R., Pollard, K.S., and Kyrpides, N.C. (2019). New insights from uncultivated genomes of the global human gut microbiome. Nature *568*, 505–510.
26. Tett, A., Huang, K.D., Asnicar, F., Fehlner-Peach, H., Pasolli, E., Karcher, N., Armanini, F., Manghi, P., Bonham, K., Zolfo, M., et al. (2019). The Prevotella copri complex comprises four distinct clades underrepresented in westernized populations. Cell Host Microbe *26*. 666.e7–679.e7.
27. Truong, D.T., Tett, A., Pasolli, E., Huttenhower, C., and Segata, N. (2017). Microbial strain-level population structure and genetic diversity from metagenomes. Genome Res. *27*, 626–638.
28. Beghini, F., McIver, L.J., Blanco-Míguez, A., Dubois, L., Asnicar, F., Maharjan, S., Mailyan, A., Thomas, A.M., Manghi, P., Valles-Colomer, M., et al. (2020). Integrating taxonomic, functional, and strain-level profiling of diverse microbial communities with bioBakery 3. eLife *10*, e65088. https://doi.org/10.1101/2020.11.19.388223.

CellPress
OPEN ACCESS

29. Chen, Q., Wu, G., Chen, H., Li, H., Li, S., Zhang, C., Pang, X., Wang, L., Zhao, L., and Shen, J. (2019). Quantification of human oral and fecal Streptococcus parasanguinis by use of quantitative real-time PCR targeting the groEL gene. Front. Microbiol. *10*, 2910.

30. Saulnier, D.M., Riehle, K., Mistretta, T.A., Diaz, M.A., Mandal, D., Raza, S., Weidler, E.M., Qin, X., Coarfa, C., Milosavljevic, A., et al. (2011). Gastrointestinal microbiome signatures of pediatric patients with irritable bowel syndrome. Gastroenterology *141*, 1782–1791.

31. Hiippala, K., Barreto, G., Burrello, C., Diaz-Basabe, A., Suutarinen, M., Kainulainen, V., Bowers, J.R., Lemmer, D., Engelthaler, D.M., Eklund, K.K., et al. (2020). Novel Odoribacter splanchnicus Strain and Its outer membrane vesicles exert immunoregulatory effects in vitro. Front. Microbiol. *11*, 575455.

32. Lee, M.J., Kang, M.J., Lee, S.Y., Lee, E., Kim, K., Won, S., Suh, D.I., Kim, K.W., Sheen, Y.H., Ahn, K., et al. (2018). Perturbations of gut microbiome genes in infants with atopic dermatitis according to feeding type. J. Allergy Clin. Immunol. *141*, 1310–1319.

33. Almeida, A., Mitchell, A.L., Boland, M., Forster, S.C., Gloor, G.B., Tarkowska, A., Lawley, T.D., and Finn, R.D. (2019). A new genomic blueprint of the human gut microbiota. Nature *568*, 499–504.

34. Almeida, A., Nayfach, S., Boland, M., Strozzi, F., Beracochea, M., Shi, Z.J., Pollard, K.S., Sakharova, E., Parks, D.H., Hugenholtz, P., et al. (2021). A unified catalog of 204,938 reference genomes from the human gut microbiome. Nat. Biotechnol. *39*, 105–114.

35. Haelewaters, D., Hofmann, T.A., and Romero-Olivares, A.L. (2021). Ten simple rules for Global North researchers to stop perpetuating helicopter research in the Global South. PLoS Comput. Biol. *17*, e1009277.

36. Abdill, R.J., Adamowicz, E.M., and Blekhman, R. (2022). Public human microbiome data are dominated by highly developed countries. PLoS Biol. *20*, e3001536.

37. Manara, S., Asnicar, F., Beghini, F., Bazzani, D., Cumbo, F., Zolfo, M., Nigro, E., Karcher, N., Manghi, P., Metzger, M.I., et al. (2019). Microbial genomes from non-human primate gut metagenomes expand the primate-associated bacterial tree of life with over 1000 novel species. Genome Biol. *20*, 299.

38. De Filippis, F., Pasolli, E., and Ercolini, D. (2020). Newly explored Faecalibacterium diversity is connected to age, lifestyle, geography, and disease. Curr. Biol. *30*, 4932–4943.e4.

39. Karcher, N., Pasolli, E., Asnicar, F., Huang, K.D., Tett, A., Manara, S., Armanini, F., Bain, D., Duncan, S.H., Louis, P., et al. (2020). Analysis of 1321 Eubacterium rectale genomes from metagenomes uncovers complex phylogeographic population structure and subspecies functional adaptations. Genome Biol. *21*, 138.

40. Schnorr, S.L., Candela, M., Rampelli, S., Centanni, M., Consolandi, C., Basaglia, G., Turroni, S., Biagi, E., Peano, C., Severgnini, M., et al. (2014). Gut microbiome of the Hadza hunter-gatherers. Nat. Commun. *5*, 3654.

41. Duranti, S., Lugli, G.A., Mancabelli, L., Armanini, F., Turroni, F., James, K., Ferretti, P., Gorfer, V., Ferrario, C., Milani, C., et al. (2017). Maternal inheritance of bifidobacterial communities and bifidophages in infants through vertical transmission. Microbiome *5*, 66.

42. Brooks, B., Olm, M.R., Firek, B.A., Baker, R., Thomas, B.C., Morowitz, M.J., and Banfield, J.F. (2017). Strain-resolved analysis of hospital rooms and infants reveals overlap between the human and room microbiome. Nat. Commun. *8*, 1814.

43. Brito, I.L., Yilmaz, S., Huang, K., Xu, L., Jupiter, S.D., Jenkins, A.P., Naisilisili, W., Tamminen, M., Smillie, C.S., Wortman, J.R., et al. (2016). Mobile genes in the human microbiome are structured from global to individual scales. Nature *535*, 435–439.

44. Parfrey, L.W., Walters, W.A., and Knight, R. (2011). Microbial eukaryotes in the human microbiome: ecology, evolution, and future directions. Front. Microbiol. *2*, 153.

45. Lukeš, J., Stensvold, C.R., Jirků-Pomajbíková, K., and Wegener Parfrey, L. (2015). Are human intestinal eukaryotes beneficial or commensals? PLoS Pathog. *11*, e1005039.

46. Laforest-Lapointe, I., and Arrieta, M.C. (2018). Microbial eukaryotes: a missing link in gut microbiome studies. mSystems *3*, https://doi.org/10.1128/mSystems.00201-17.

47. Beghini, F., Pasolli, E., Truong, T.D., Putignani, L., Cacciò, S.M., and Segata, N. (2017). Large-scale comparative metagenomics of Blastocystis, a common member of the human gut microbiome. ISME J. *11*, 2848–2863.

48. Scanlan, P.D., Hill, C.J., Ross, R.P., Ryan, C.A., Stanton, C., and Cotter, P.D. (2018). The intestinal protist Blastocystis is not a common member of the healthy infant gut microbiota in a Westernized country (Ireland). Parasitology *145*, 1274–1278.

49. Gebremariam, M.M., Zarnkow, M., and Becker, T. (2014). Teff (Eragrostis tef) as a raw material for malting, brewing and manufacturing of gluten-free foods and beverages: a review. J. Food Sci. Technol. *51*, 2881–2895.

50. Moroni, A.V., Arendt, E.K., and Dal Bello, F. (2011). Biodiversity of lactic acid bacteria and yeasts in spontaneously-fermented buckwheat and teff sourdoughs. Food Microbiol. *28*, 497–502.

51. Tilahun, B., Tesfaye, A., Muleta, D., Bahiru, A., Terefework, Z., and Wessel, G. (2018). Isolation and Molecular Identification of lactic acid Bacteria Using 16S rRNA Genes from Fermented Teff (Eragrostis tef (Zucc.)) Dough. Int. J. Food Sci. *2018*, 8510620.

52. Tadesse, B.T., Abera, A.B., Tefera, A.T., Muleta, D., Alemu, Z.T., and Wessel, G. (2019). Molecular characterization of fermenting yeast species from fermented Teff dough during preparation of injera using ITS DNA sequence. Int. J. Food Sci. *2019*, 1291863.

53. Ua-Arak, T., Jakob, F., and Vogel, R.F. (2016). Characterization of growth and exopolysaccharide production of selected acetic acid bacteria in buckwheat sourdoughs. Int. J. Food Microbiol. *239*, 103–112.

54. De Vuyst, L., Van Kerrebroeck, S., and Leroy, F. (2017). Microbial ecology and process technology of sourdough fermentation. Adv. Appl. Microbiol. *100*, 49–160.

55. Tamang, J.P., Cotter, P.D., Endo, A., Han, N.S., Kort, R., Liu, S.Q., Mayo, B., Westerik, N., and Hutkins, R. (2020). Fermented foods in a global age: east meets West. Compr. Rev. Food Sci. Food Saf. *19*, 184–217.

56. Pasolli, E., De Filippis, F., Mauriello, I.E., Cumbo, F., Walsh, A.M., Leech, J., Cotter, P.D., Segata, N., and Ercolini, D. (2020). Large-scale genome-wide analysis links lactic acid bacteria from food with the gut microbiome. Nat. Commun. *11*, 2610.

57. Tamburini, F.B., Maghini, D., Oduaran, O.H., Brewster, R., Hulley, M.R., Sahibdeen, V., Norris, S.A., Tollman, S., Kahn, K., Wagner, R.G., et al. (2022). Short- and long-read metagenomics of urban and rural South African gut microbiomes reveal a transitional composition and undescribed taxa. Nat. Commun. *13*, 926.

58. de Goffau, M.C., Jallow, A.T., Sanyang, C., Prentice, A.M., Meagher, N., Price, D.J., Revill, P.A., Parkhill, J., Pereira, D.I.A., and Wagner, J. (2022). Gut microbiomes from Gambian infants reveal the development of a non-industrialized Prevotella-based trophic network. Nat. Microbiol. *7*, 132–144.

59. El Safadi, D., Gaayeb, L., Meloni, D., Cian, A., Poirier, P., Wawrzyniak, I., Delbac, F., Dabboussi, F., Delhaes, L., Seck, M., et al. (2014). Children of Senegal River Basin show the highest prevalence of Blastocystis sp. ever observed worldwide. BMC Infect. Dis. *14*, 164.

60. Clark, C.G., van der Giezen, M., Alfellani, M.A., and Stensvold, C.R. (2013). Recent developments in Blastocystis research. Adv. Parasitol. *82*, 1–32.

61. Cinek, O., Polackova, K., Odeh, R., Alassaf, A., Kramná, L., Ibekwe, M.U., Majaliwa, E.S., Ahmadov, G., Elmahi, B.M.E., Mekki, H., et al. (2021). Blastocystis in the faeces of children from six distant countries: prevalence, quantity, subtypes and the relation to the gut bacteriome. Parasit. Vectors *14*, 399.

62. Milani, C., Duranti, S., Napoli, S., Alessandri, G., Mancabelli, L., Anzalone, R., Longhi, G., Viappiani, A., Mangifesta, M., Lugli, G.A., et al. (2019).

Colonization of the human gut by bovine bacteria present in Parmesan cheese. Nat. Commun. *10*, 1286.

63. Peluzio, M.d.C.G., Dias, M.d.M.E., Martinez, J.A., and Milagro, F.I. (2021). Kefir and intestinal microbiota modulation: implications in human health. Front. Nutr. *8*, 638740. https://doi.org/10.3389/fnut.2021.638740.
64. Mengesha, Y., Tebeje, A., and Tilahun, B. (2022). A review on factors influencing the fermentation process of teff (eragrostis teff) and other cereal-based ethiopian injera. Int. J. Food Sci. *2022*, 4419955.
65. Li, D., Liu, C.M., Luo, R., Sadakane, K., and Lam, T.W. (2015). MEGAHIT: an ultra-fast single-node solution for large and complex metagenomics assembly via succinct de Bruijn graph. Bioinformatics *31*, 1674–1676.
66. Kang, D.D., Li, F., Kirton, E., Thomas, A., Egan, R., An, H., and Wang, Z. (2019). MetaBAT 2: an adaptive binning algorithm for robust and efficient genome reconstruction from metagenome assemblies. PeerJ *7*, e7359.
67. Parks, D.H., Imelfort, M., Skennerton, C.T., Hugenholtz, P., and Tyson, G.W. (2015). CheckM: assessing the quality of microbial genomes recovered from isolates, single cells, and metagenomes. Genome Res. *25*, 1043–1055.
68. Asnicar, F., Thomas, A.M., Beghini, F., Mengoni, C., Manara, S., Manghi, P., Zhu, Q., Bolzan, M., Cumbo, F., May, U., et al. (2020). Precise phylogenetic analysis of microbial isolates and genomes from metagenomes using PhyloPhlAn 3.0. Nat. Commun. *11*, 2500.
69. Ondov, B.D., Treangen, T.J., Melsted, P., Mallonee, A.B., Bergman, N.H., Koren, S., and Phillippy, A.M. (2016). Mash: fast genome and metagenome distance estimation using MinHash. Genome Biol. *17*, 132.
70. Stamatakis, A. (2014). RAxML version 8: a tool for phylogenetic analysis and post-analysis of large phylogenies. Bioinformatics *30*, 1312–1313. https://doi.org/10.1093/bioinformatics/btu033.
71. Li, H., Handsaker, B., Wysoker, A., Fennell, T., Ruan, J., Homer, N., Marth, G., Abecasis, G., and Durbin, R.; 1000 Genome Project Data Processing Subgroup (2009). The Sequence Alignment/Map format and SAMtools. Bioinformatics *25*, 2078–2079.
72. Quinlan, A.R. (2014). BEDTools: the Swiss-army tool for genome feature analysis. Curr. Protoc. Bioinformatics *47*, 11.12.1–11.1234.
73. Langmead, B., and Salzberg, S.L. (2012). Fast gapped-read alignment with Bowtie 2. Nat. Methods *9*, 357–359.
74. Oksanen, J., Kindt, R., Legendre, P., O'Hara, B., Henry, M., and Stevens, M.H.H. (2007). The vegan package. Community Ecol. Package *10*, 719.
75. Wickham, H. (2016). ggplot2: Elegant Graphics for Data Analysis (Springer).
76. O'Leary, N.A., Wright, M.W., Brister, J.R., Ciufo, S., Haddad, D., McVeigh, R., Rajput, B., Robbertse, B., Smith-White, B., Ako-Adjei, D., et al. (2016). Reference sequence (RefSeq) database at NCBI: current status, taxonomic expansion, and functional annotation. Nucleic Acids Res. *44*, D733–45. https://doi.org/10.1093/nar/gkv1189.
77. Overview World Bank. The World Bank in Ethiopia, 2022. https://www.worldbank.org/en/country/ethiopia/overview.
78. Haar, S., and Hassen, M. (1992). The Oromo of Ethiopia: a history, 1570–1860. Ethnohistory *39*, 88. https://doi.org/10.2307/482578.
79. Fetene, N., Linnander, E., Fekadu, B., Alemu, H., Omer, H., Canavan, M., Smith, J., Berman, P., and Bradley, E. (2016). The Ethiopian health extension program and variation in health systems performance: what matters? PLoS One *11*, e0156438.
80. Lock, M.M. (1994). Encounters with Aging: Mythologies of Menopause in Japan and North America (University of California Press).
81. Niewöhner, J., and Lock, M. (2018). Situating local biologies: anthropological perspectives on environment/human entanglements. BioSocieties *13*, 681–697.
82. Amato, K.R., Arrieta, M.C., Azad, M.B., Bailey, M.T., Broussard, J.L., Bruggeling, C.E., Claud, E.C., Costello, E.K., Davenport, E.R., Dutilh, B.E., et al. (2021). The human gut microbiome and health inequities. Proc. Natl. Acad. Sci. USA *118*, https://doi.org/10.1073/pnas.2017947118.
83. Benezra, A. (2020). Race in the microbiome. Sci. Technol. Hum. Values *45*, 877–902.
84. Greenhough, B., Read, C.J., Lorimer, J., Lezaun, J., McLeod, C., Benezra, A., Bloomfield, S., Brown, T., Clinch, M., D'Acquisto, F., et al. (2020). Setting the agenda for social science research on the human microbiome. Palgrave Commun. *6*, 1–11.
85. Ishaq, S.L., Parada, F.J., Wolf, P.G., Bonilla, C.Y., Carney, M.A., Benezra, A., Wissel, E., Friedman, M., DeAngelis, K.M., Robinson, J.M., et al. (2021). Introducing the microbes and social equity working group: considering the microbial components of social, environmental, and health justice. mSystems *6*, e0047121. https://doi.org/10.1128/mSystems.00471-21.
86. Ishaq, S.L., Wissel, E.F., Wolf, P.G., Grieneisen, L., Eggleston, E.M., Mhuireach, G., Friedman, M., Lichtenwalner, A., Otero Machuca, J., Weatherford Darling, K., et al. (2022). Designing the Microbes and Social Equity Symposium: a Novel Interdisciplinary Virtual Research Conference Based on Achieving Group-Directed Outputs. Challenges *13*, 30.
87. Reardon, J. (2018). The Postgenomic Condition: Ethics, Justice, and Knowledge after the Genome (University of Chicago Press). jereardon.sites.ucsc.edu.
88. Robinson, J.M., Redvers, N., Camargo, A., Bosch, C.A., Breed, M.F., Brenner, L.A., Carney, M.A., Chauhan, A., Dasari, M., Dietz, L.G., et al. (2022). Twenty important research questions in microbial exposure and social equity. mSystems *7*, e0124021.
89. Human Microbiome Project Consortium (2012). Structure, function and diversity of the healthy human microbiome. Nature *486*, 207–214.
90. Bowers, R.M., Kyrpides, N.C., Stepanauskas, R., Harmon-Smith, M., Doud, D., Reddy, T.B.K., Schulz, F., Jarett, J., Rivers, A.R., Eloe-Fadrosh, E.A., et al. (2017). Minimum information about a single amplified genome (MISAG) and a metagenome-assembled genome (MIMAG) of bacteria and archaea. Nat. Biotechnol. *35*, 725–731.
91. Li, H., and Durbin, R. (2009). Fast and accurate short read alignment with Burrows-Wheeler transform. Bioinformatics *25*, 1754–1760.

STAR★METHODS

KEY RESOURCES TABLE

REAGENT or RESOURCE	SOURCE	IDENTIFIER
**Critical commercial assays**		
PowerSoil DNA Isolation Kit	QIAGEN, Germany	Catalog No. 12888-50
NexteraXT DNA Library Preparation Kit	Illumina, California, USA	FC-131-1096
**Deposited data**		
Previously sequenced cohorts in Ethiopia, Ghana, Tanzania	Tett et al.[26]	NCBI-SRA BioProject: PRJNA504891; PRJNA529124; PRJNA529400
Previously sequenced cohorts in Italy	Asnicar et al.[15]; Ferretti et al.[16]	NCBI-SRA BioProject: PRJNA352475
Previously sequenced cohort in Sweden	Bäckhed et al.[10]	EBI's Sequence Read Archive: ERP005989
Newly sequenced cohort in Italy	This study	NCBI-SRA BioProject: PRJNA352475 and PRJNA716780
Newly sequenced teff sourdough metagenomes	This study	NCBI-SRA BioProject: PRJNA504891
**Software and algorithms**		
MEGAHIT (version 1.1.1)	Li et al.[65]	RRID: SCR_018551; https://github.com/voutcn/megahit
MetaBAT2 (version 2.12.1)	Kang et al.[66]	RRID: SCR_019134; https://bitbucket.org/berkeleylab/metabat
CheckM (version 1.0.7)	Parks et al.[67]	RRID: SCR_016646; https://github.com/Ecogenomics/CheckM
CMSeq (version 1.0.0)	Pasolli et al.[23]	https://bitbucket.org/CibioCM/cmseq
PhyloPhlAn 3 (version 3.0.2)	Asnicar et al.[68]	https://github.com/biobakery/phylophlan
Mash (version 2.0)	Ondov et al.[69]	RRID: SCR_019135; https://github.com/marbl/Mash
MetaPhlAn 3 (version 3.0)	Beghini et al.[28]	https://github.com/biobakery/MetaPhlAn
StrainPhlAn 3 (version 3.0)	Beghini et al.[28]	https://github.com/biobakery/MetaPhlAn
RAxML (version 8.1.15)	Stamatakis[70]	RRID: SCR_006086; https://github.com/stamatak/standard-RAxML
SAMtools (version 1.3.1)	Li et al.[71]	RRID: SCR_002105; https://github.com/samtools/samtools
BEDtools	Quinlan[72]	RRID: SCR_006646; https://github.com/arq5x/bedtools2
bowtie2 (version 2.4.0)	Langmead and Salzberg[73]	RRID: SCR_016368; https://github.com/BenLangmead/bowtie2
Vegan R package	Oksanen et al.[74]	RRID: SCR_011950; https://github.com/vegandevs/vegan
ggplot2 (version 3.3.2)	Wickham[75]	RRID: SCR_014601; https://github.com/tidyverse/ggplot2
**Other**		
RefSeq (F. sanfranciscensis genomes)	O'Leary et al. 2016[76]	RRID: SCR_003496; https://www.ncbi.nlm.nih.gov/refseq/

RESOURCE AVAILABILITY

Lead contact

Further information and requests for resources and reagents should be directed to and will be fulfilled by the lead contact, Nicola Segata (nicola.segata@unitn.it).

Materials availability

This study did not generate new unique reagents.

Data and code availability

- Raw data for the 72 metagenomes newly sequenced in this study are publicly available in NCBI-SRA as of the date of publication. Accession numbers are listed in the key resources table. This paper analyzes existing, publicly available data. These accession numbers for the datasets are listed in the key resources table.
- All original code has been deposited and is publicly available as of the date of publication. DOIs are listed in the key resources table.

- Any additional information required to reanalyze the data reported in this paper is available from the lead contact upon request.

EXPERIMENTAL MODEL AND SUBJECT DETAILS

Mother-infant cohorts surveyed in this study

To expose lifestyle-dependent biases in the rate and species-specificity of mother-infant strain sharing events, we included stool metagenomes obtained from an Ethiopian cohort we previously sequenced,[26] one Swedish,[10] and two Italian[15,16] mother-infant cohorts. We moreover included 70 newly sequenced follow-up samples from this last cohort and from another cohort of healthy newborns and children aged 0-11 years and their mothers living in urban areas in Northern Italy (Table S4). As representatives of other non-Westernized mother-infant cohorts, we selected mother-infant pairs from Ghana and Tanzania[26] with infants of the same age range as Ethiopian ones (newborns, here defined as infants <1 year, and children, here defined as infants aged 1-12 years). Infants were vaginally delivered with no perinatal antibiotic prophylaxis exposure, except for some C-section delivered Italian (15/156 Italian samples) and Swedish newborns (30/300 Swedish samples). In addition to stool microbiomes from mothers and infants, we moreover collected and sequenced two samples of teff sourdough from one fermentation pot (Table S4). Teff is used in this community to prepare different foods, including injera, a flatbread made of teff flour and water fermented for 24 h and steam-cooked for 1–3 min on a gentle heat, or genfo, a kind of teff-based porridge served with a tablespoon of clarified butter and paprika. In the community, genfo is traditionally consumed by new mothers after birth to regain energy, and it is served to weaning babies. Households share the same food bowl, and the family consumes food in a patriarchal order, with men eating first, followed by male children, female children, and women. Women are also in charge of cooking for the household and prepare, ferment, and cook teff-based sourdough, therefore being exposed to the sourdough microbes while at the same time representing a source of microbes for the sourdough. In total, 702 metagenomes were included in the study.

Lifestyle of the Ethiopian communities assessed in this study

Ethiopia is the largest and most populated country in the Horn of Africa. Ethiopia is the second most populous nation in Africa after Nigeria, and still the fastest growing economy in the region, with 6.3 percent growth in FY2020/21. However, it is also one of the poorest, with a per capita gross national income of $890.[77] Gimbichu and Igu-kura are two towns at the core of Ethiopia, about 100 kms from the capital Addis Ababa and the homeland of the Oromo people.[78] In these towns, the typical household is a hut made of cow droppings and mud, with a straw roof and an earthen floor. The cabin consists of a single space, where people cook with firewood and live with the animals; the spaces are barely separated with a cloth or blanket, as a common bedroom, and the family sleeps on straw or blanket, on the floor or cot, all together. The animals enter and leave the enclosure freely and at night they sleep inside the enclosure.

In the huts, there is no running water, electricity, or latrines. The wells that have been drilled in these towns have improved the health and quality of life of the community, especially the boys and girls who suffered and still suffer, to a lesser extent, from parasites.

To access healthcare, people of the community can refer to the nearest hospital, but the costs of transportation, food, treatments, and accommodation or hospitalization must be paid for by the patient or his family. There are not many biomedical specialists available. Because of this, people of these communities do not usually leave their work in the fields or at home to go to the hospital, but they resort to the medicine of local healers first. Access to public healthcare is for those who have resources, but those who have resources prefer private healthcare.[79]

It is a subsistence economy, based on crops, which are usually teff, the cereal with which they prepare the enjera, a basic and essential fermented food, to which they add potatoes, chickpeas, broad beans and/or lentils, which are also grown in the area. During the celebrations they prepare bread as a special food, which they combine with a kind of paprika called "mirmita", and they offer popcorn and coffee. Both coffee and enjera are consumed daily, if the generally precarious economy of the family allows it. Carbonated drinks begin to appear at celebrations, which they consider a sign of their entering in a globalized economy and society.

In the capital and more urban centers, Westernized lifestyles and eating habits are taking hold and the inhabitants of these cities consume a significant amount of carbohydrates and simple sugars such as: bread, pasta, rice, cookies, soluble cocoa, sugar drinks etc. This way of eating affects an increase in noncommunicable inflammatory diseases such as type II diabetes and obesity.

Definition of Westernized and Non-Westernized lifestyles

The microbiome reflects lifestyle which varies not only between but also within human populations. In anthropology, it has been established that biology is always "situated", or rather, influenced by socio-political and material conditions.[80] Nowadays, these conditions are articulated in different ways in the encounter with global processes[81] and populations are highly differentiated and dynamic and bio-social diversity cannot be encapsulated into homogenous population labels.

In this work, we define populations with Westernized lifestyles as those cohorts living in highly industrialized countries, with limited contact with wildlife, high-calorie diets, higher exposure to xenobiotics, highly processed foods, antibiotics, and antimicrobials. Populations living in more rural areas with larger exposure to wildlife or domesticated animals, local food production and consumption, and limited access to pharmaceuticals, are here defined as cohorts with a non-Westernized lifestyle. According to these definitions, in this work, we consider the available Italian cohorts,[15,16] the newly sequenced Italian cohort, and the Swedish cohort[10] as coming from Westernized populations, and the Ghanaian, Tanzanian, and Ethiopian[26] cohorts as coming from non-Westernized populations. Variation

across and within populations does not depend solely on lifestyle patterns as these are crucially grounded in socio-political circumstances such as access to healthcare and education, socio-economic status, market integration, degree of industrialization, pollution, housing, infrastructure, etc.[82–88] that cut across and intersect the individual to the regional, national and macro-geographical scale.

METHOD DETAILS

Newly sequenced metagenomes

In addition to the published metagenomes reported in the previous paragraph, we included in this study 70 newly sequenced samples from healthy Italian mothers and infants and two samples of fermented teff-dough collected in the Ethiopian community previously sequenced in Tett et al.[26] Fourteen newly sequenced stool samples were later time points of infants belonging to the cohort previously described in Ferretti et al. [16], namely stool samples collected at 7, 12, and 24 months of the infants (Table S4). DNA was extracted with the PowerSoil DNA Isolation Kit (Qiagen)[89] and libraries were constructed with the NexteraXT DNA Library Preparation Kit (Illumina) and sequenced on the Illumina HiSeq2500 100nt paired end platform (target depth: 5Gb/sample). Protocols were approved by the Ethics Committee of the University of Trento and the Ethics Committee of Santa Chiara Hospital (Trento, Italy, EC ref number: 51082283 - 30/07/2014), as previously reported.[16] The remaining 56 newly sequenced stool metagenomes were instead collected from healthy mothers and infants (Table S4) at the IRCCS Istituto Giannina Gaslini, Genoa, Italy, after ethical approval by the Regional Ethics Committee of Liguria (N° 006/2019). Collection of the fermented teff dough was performed in agreement with the protocols approved by the Ethics Committee of the Consejo Superior de Investigaciones Cientìficas (Madrid, Spain, nr. 058/2018) and by the Research Ethics Committee of the Valencia University (ref. nr. H1484811493170).

MAGs reconstruction, clustering, and taxonomic assignment

Metagenome-assembled genomes (MAGs) were reconstructed through single-sample metagenomic assembly and contig binning as validated elsewhere.[23] Briefly, assemblies were produced with MEGAHIT,[65] and MetaBAT2[66] was used to bin contigs longer than 1,000 nt. This approach produced 11,250 medium- and high-quality MAGs with CheckM 1.0.7[67] completeness >50% and contamination <5%, as defined in Bowers et al.[90] MAGs were clustered into species-level genome bins (SGBs), genus-level genome bins (GGBs), and family-level genome bins (FGBs) spanning 5%, 15%, and 30% of genetic diversity respectively, as previously described in Pasolli et al.[23] The SGB assignment is based on the application of a subroutine of PhyloPhlAn 3 called 'phylophlan_metagenomic' which makes use of MASH[69] to compute the average genetic distance between genomes and SGBs. A genome was assigned to the closest SGB in case its genetic distance resulted below 5%. Otherwise, new SGBs were defined by applying a hierarchical clustering based on the average linkage. The same procedure was applied for the assignment and definition of new GGBs and FGBs at 15% and 30% of genetic distance. Every genome finally inherited a taxonomic label from the assigned known or unknown SGB, called kSGB and uSGB respectively, whose difference resides in the presence and absence of reference genomes. Taxonomies of kSGBs were defined by applying a majority voting mechanism on the taxonomic labels of their reference genomes. In case of SGBs with no reference genomes (uSGBs), a taxonomic label was inferred by applying the same mechanism at the GGB level. The same logic was repeated by considering the reference genomes at the FGB level in case of uGGBs (GGBs with no reference genomes). Ultimately, in case of uFGBs, a taxonomic label was assigned by considering up to 100 closest reference genomes reported by 'phylophlan_metagenomic'.

Species-level and strain-level profiling of mother-infant cohorts

The relative abundance of known microbial species was estimated using MetaPhlAn 3[28] version 3.0 with marker database version 201901.

Strain-level profiling of SGBs was performed with MetaPhlAn 3 and StrainPhlAn 3[28] using a custom marker database including a collection of 17,340 SGBs previously published,[23,37] with the addition of the 146 new uSGBs recovered in this study. Unknown SGBs with less than 5 MAGs overall (including MAGs external from the study cohorts) were excluded, as we cannot ensure they are not populated with assembly artifacts or chimeric genomes. The core genes of each SGB were divided into 150 nucleotide fragments and aligned against the genomes of all SGBs using bowtie2 (version 2.3.5.1; –sensitive option).[73] A core gene was considered present in a genome if at least one of the gene's fragments was mapped against it. Core genes never found in more than 1% of the sequences included in any other SGBs were selected as marker genes.

To infer strain sharing, strain-level phylogenies were then reconstructed using MetaPhlAn 3 and StrainPhlAn3 with parameters "–marker_in_n_samples 5 –sample_with_n_markers 10—phylophlan_mode accurate". For detecting strain sharing events, we executed the StrainPhlAn's strain_transmission.py script with default parameters. Using the StrainPhlAn's phylogenetic trees, this script generates a pairwise distance matrix normalized by the total branch length of the tree and infers a threshold defining identical strains by selecting the first percentile of the distribution of the non-related-samples distances. Related samples were defined as samples coming from the same subject or mother-infant pairs. SGBs with a distance smaller than the inferred threshold are reported as strain-sharing events.

Phylogenetic analysis of the SGBs shared across all infants cohorts

Phylogenetic trees of the five selected SGBs (IDs: 1814, 1934, 4290, 4285,4933, 15316, 15318) were reconstructed with PhyloPhlAn 3.0[68] with the following parameters: "–diversity low –fast –trim greedy –min_num_marker [50%]". Mapping was performed with

BLAST with parameters "-outfmt 6 -max_target_seqs 1000000", MSA was performed with MAFFT with parameters "–localpair –maxiterate 1000 –anysymbol", and the phylogenetic tree was reconstructed with RAxML with parameters "-m GTRCAT". Custom SGB-specific databases have been constructed with the "phylophlan_setup_database" script with the core genes identified as described in the "Species-level and strain-level profiling of mother-infant cohorts STAR Methods section.

Blastocystis sp. profiling and SNP calling

We identified *Blastocystis* sp. by applying previously validated methods and thresholds.[47] Briefly, metagenomic reads were mapped against reference genomes that included ST1, ST2, ST3, ST4, ST6, ST7, ST8, and ST9 (Table S2) using Bowtie2.[73] Bowtie2 output was processed with SAMtools[71] and BEDtools[72] to obtain the breadth of coverage for each ST (parameters "genomecov –bg"). Only samples with a breadth of coverage >10% for a specific ST reference genome were considered positive for *Blastocystis* sp. and for that specific ST (Table S2).

We sought to analyze strain sharing events between family members for ST1, ST2, ST3, ST4, and ST6, combining reference-based mapping approach and phylogenetic analysis. Processed metagenomic data were first aligned against reference genomes representative of five *Blastocystis* subtypes respectively, using bowtie2[73] with parameters "–end-to-end -a". The resulting bam files were used to reconstruct, based on each reference genome, multiple sequence alignments (MSAs) comprising strain consensus sequences of mapped metagenomic reads using a python package CMSeq (https://github.com/SegataLab/cmseq) with following criteria: (1) mapping quality >= 30, (2) coverage >= 5 folds, (3) minimum identity of reads >= 97%, (4) aligned read length >= 30nt, (5) minimum dominant allele frequency >= 80%. Next, we excluded columns containing > 50% missing data in each MSA, and performed phylogenetic analysis on the cleaned MSAs using RAxML (v8.1.15)[70] under a GTR model of substitution with 4 gamma categories and 100 bootstrap pseudoreplicates.

Boxplots and heatmaps were produced with ggplot2 v. 3.3.2.[75]

Fructilactobacillus sanfranciscensis (previously *Lactobacillus sanfranciscensis*) mapping

As the coverage was too low to perform StrainPhlAn profiling, we downloaded all the *F. sanfranciscensis* genomes (n = 31) available in NCBI RefSeq[76] as December 2020 and integrated them with the two MAGs of the same species reconstructed from injera. We mapped raw reads against this set of 33 genomes/MAGs with bowtie2 (v. 2.4.0, "–sensitive" option)[73] and computed the breadth of coverage using SAMtools (v. 1.3.1).[91]

QUANTIFICATION AND STATISTICAL ANALYSIS

Non-metric multidimensional scaling plots were generated using the Bray-Curtis distance on sample taxonomic composition with the metaMDS function in the vegan R package[74] and visualized with ggplot2 v. 3.3.2..[75] Permutational analysis of variance (PERMANOVA) was performed with the adonis function in the vegan R package.[74]

Statistical significance was assessed through Fisher, Wilcoxon or Mann-Whitney U's tests with multiple hypothesis testing Benjamini-Hochberg FDR corrections, as reported in the text. Because of the different age distribution between Ethiopian and Westernized infants, we randomly picked Westernized infants aged 0–6 months, 6-12 months, and 1-12 years to get a similar age distribution with respect to Ethiopian ones before performing statistical tests. Each test was repeated 10 times with 10 different random pickings, with each test comparing the microbiome composition of 127 Westernized and 20 Ethiopian infants (0-6 mo.: 55 West. and 12 Eth.; 6-12 mo.: 27 West. and 6 Eth.; 12-36 mo.: 9 West. and 2 Eth.). Benjamini-Hochberg FDR corrections were performed to account for multiple hypothesis testing. Non-Westernized cohorts already had an age distribution similar to the Ethiopian one.

Current Biology, Volume *33*

Supplemental Information

Maternal and food microbial sources shape the infant microbiome of a rural Ethiopian population

Serena Manara, Marta Selma-Royo, Kun D. Huang, Francesco Asnicar, Federica Armanini, Aitor Blanco-Miguez, Fabio Cumbo, Davide Golzato, Paolo Manghi, Federica Pinto, Mireia Valles-Colomer, Loredana Amoroso, Maria Valeria Corrias, Mirco Ponzoni, Roberta Raffaetà, Raul Cabrera-Rubio, Mari Olcina, Edoardo Pasolli, Maria Carmen Collado, and Nicola Segata

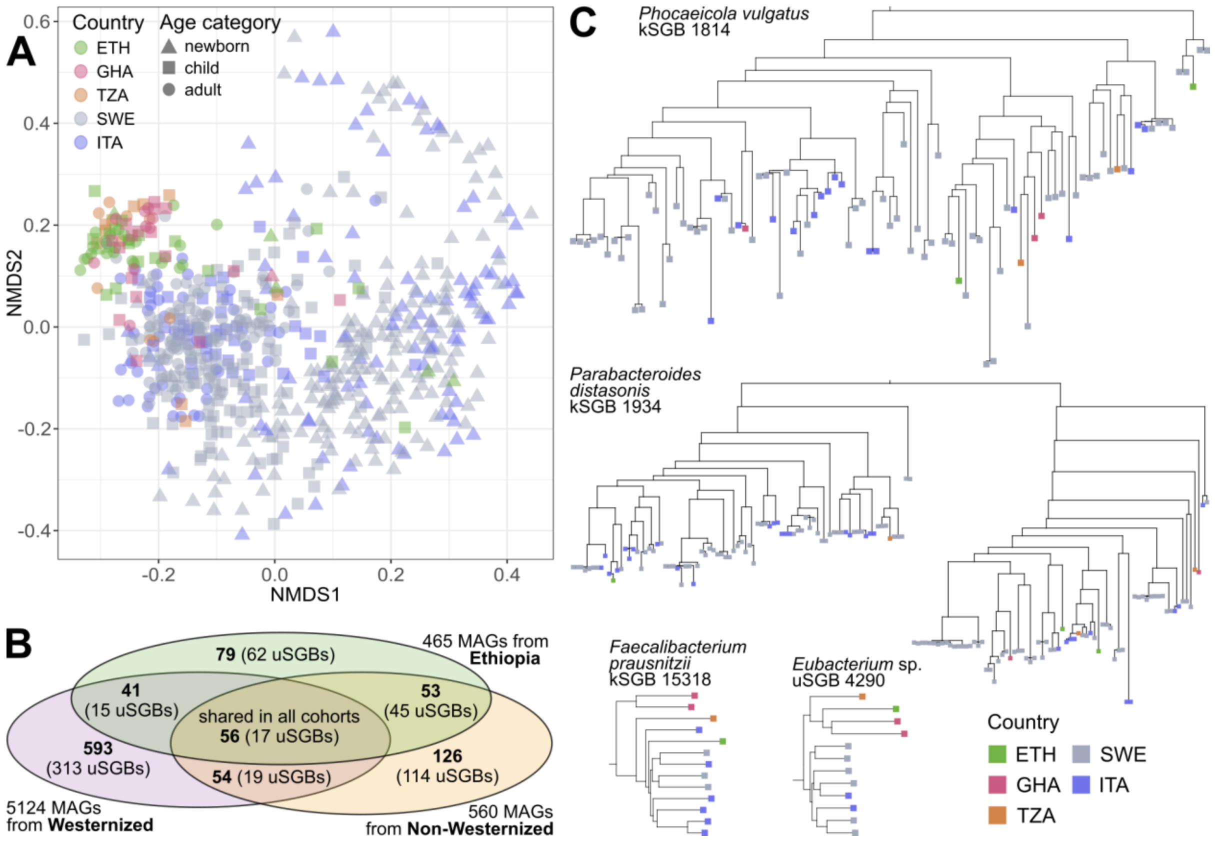

Figure S1. Ethiopian mother and infant microbiome composition differs from that of Italian and Swedish age-matched populations and is more similar to the one of Tanzanian and Ghanaian ones. Related to Figure 1. A) Multidimensional scaling of the Bray-Curtis distance on sample taxonomic composition shows a trend ranging from newborns (triangles, <1 year) to children (squares, >1 year) and adults (circles, >18 years). Samples with single species relative abundance > 90% have been removed. **B)** SGB sharing across different infant cohorts. Differently from what is reported in **Figure 1C**, here an SGB is considered shared between Ethiopia and non-Westernized (or Westernized) cohorts even if it is present only in one of the two countries part of that specific lifestyle category. Even in this case, Ethiopian infants share a larger proportion of SGBs and particularly uSGBs with infants from other Non-Westernized cohorts (26.7% of the total number of SGBs found in the Ethiopian and non-Westernized infants) and have an even more limited SGB overlap with Westernized infants microbiomes (11.1% of of the total number of SGBs found in the Ethiopian and Westernized infants). **C)** Phylogenetic trees of additional relevant species shared across Ethiopian, Westernized, and Non-Westernized infants, including two further SGBs of the same species reported in **Figure 1D**.

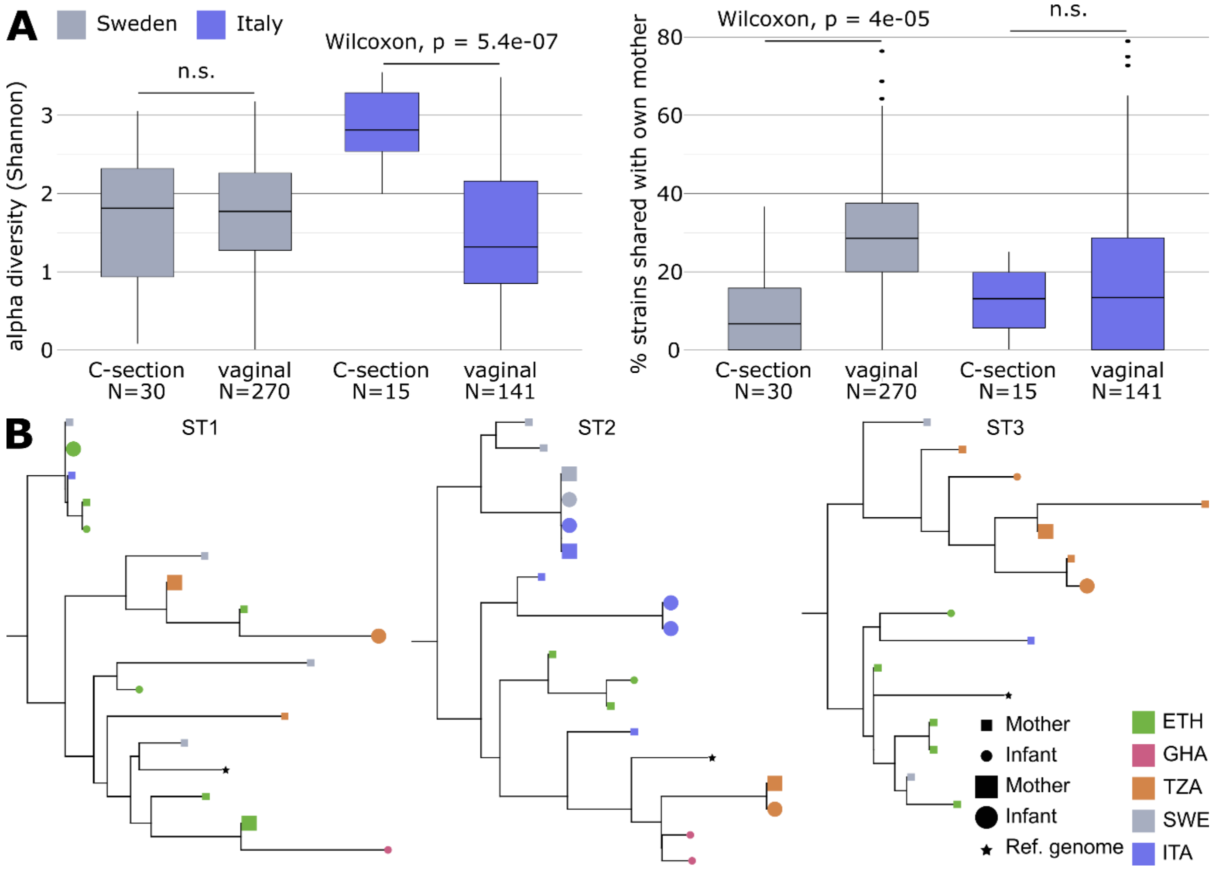

Figure S2. Potential modifiers of strain-sharing rate. Related to Figures 2 and 3. A) Differences in alpha diversity (Shannon) and strain sharing with own mother by mode of delivery for the two cohorts that include samples from C-section delivered infants (Sweden 30/300, Italy 15/156). **B)** Phylogenetic trees of *Blastocystis* sp. STs present also in Ethiopian samples (ST1, ST2, ST3). ST2 shows the highest degree of mother-infant sharing, with 4 out of 4 positive pairs showing the presence of the same strain. ST1 and ST3, instead, were not shared at the strain level between mother and infant.
